# Imaging in rhabdomyosarcoma: a patient journey

**DOI:** 10.1007/s00247-023-05596-8

**Published:** 2023-02-27

**Authors:** Isabelle S. A. de Vries, Roelof van Ewijk, Laura M. E. Adriaansen, Anneloes E. Bohte, Arthur J. A. T. Braat, Raquel Dávila Fajardo, Laura S. Hiemcke-Jiwa, Marinka L. F. Hol, Simone A. J. ter Horst, Bart de Keizer, Rutger R. G. Knops, Michael T. Meister, Reineke A. Schoot, Ludi E. Smeele, Sheila Terwisscha van Scheltinga, Bas Vaarwerk, Johannes H. M. Merks, Rick R. van Rijn

**Affiliations:** 1 Princess Máxima Center for Pediatric Oncology, Utrecht, the Netherlands; 2 Department of Radiology and Nuclear Medicine, University Medical Centre Utrecht, Utrecht, the Netherlands; 3 Department of Radiotherapy, University Medical Centre Utrecht, Utrecht, the Netherlands; 4 Department of Pathology, University Medical Centre Utrecht, Utrecht, the Netherlands; 5 Department of Otorhinolaryngology, University Medical Centre Utrecht, Utrecht, the Netherlands; 6 Oncode Institute, Utrecht, the Netherlands; 7 Department of Head and Neck Oncology and Surgery, The Netherlands Cancer Institute (NCI), Amsterdam, the Netherlands; 8 Department of Oral and Maxillofacial Surgery, Amsterdam UMC, University of Amsterdam, Amsterdam, the Netherlands; 9 Department of Paediatrics, Amsterdam UMC – Emma Children’s Hospital, University of Amsterdam, Amsterdam, the Netherlands; 10 Department of Radiology and Nuclear Medicine, Amsterdam UMC – Emma Children’s Hospital, University of Amsterdam, Suite C1-423.1, Meibergdreef 9, 1105AZ Amsterdam, the Netherlands

**Keywords:** Adolescent, Child, Medical oncology, Radiology, Rhabdomyosarcoma

## Abstract

Rhabdomyosarcoma, although rare, is the most frequent soft tissue sarcoma in children and adolescents. It can present as a mass at nearly any site in the body, with most common presentations in the head and neck, genitourinary tract and extremities. The optimal diagnostic approach and management of rhabdomyosarcoma require a multidisciplinary team with multimodal treatment, including chemotherapy and local therapy. Survival has improved over the last decades; however, further improvement in management is essential with current 5-year overall survival ranging from 35% to 100%, depending on disease and patient characteristics. In the full patient journey, from diagnosis, staging, management to follow-up after therapy, the paediatric radiologist and nuclear physician are essential members of the multidisciplinary team. Recently, guidelines of the European paediatric Soft tissue sarcoma Study Group, the Cooperative Weichteilsarkom Studiengruppe and the Oncology Task Force of the European Society of Paediatric Radiology (ESPR), in an ongoing collaboration with the International Soft-Tissue Sarcoma Database Consortium, provided guidance for high-quality imaging. In this educational paper, given as a lecture during the 2022 postgraduate ESPR course, the multi-disciplinary team of our national paediatric oncology centre presents the journey of two patients with rhabdomyosarcoma and discusses the impact on and considerations for the clinical (paediatric) radiologist and nuclear physician. The key learning points of the guidelines and their implementation in clinical practice are highlighted and up-to-date insights provided for all aspects from clinical suspicion of rhabdomyosarcoma and its differential diagnosis, to biopsy, staging, risk stratification, treatment response assessment and follow-up.

## Introduction

**Fig. 1:**
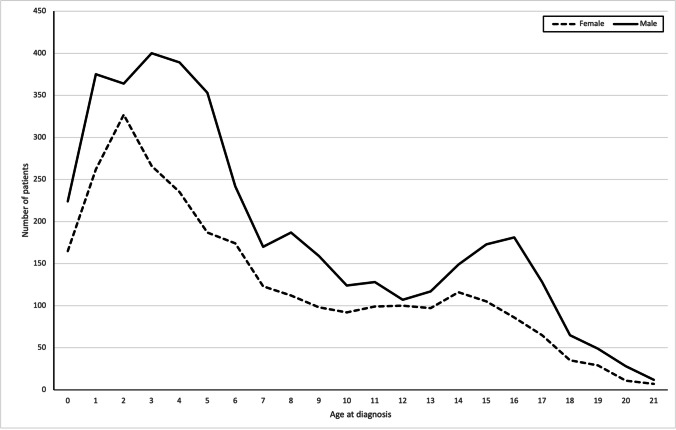
Age distribution at diagnosis, based on data registered in the International Soft Tissue Sarcoma Consortium’s database (*n* = 6,961 patients) [2]. Not shown is data of 46 patients over the age of 21 years (14 female, ages 22–41, and 32 male, ages 22–45 years)

**Fig. 2:**
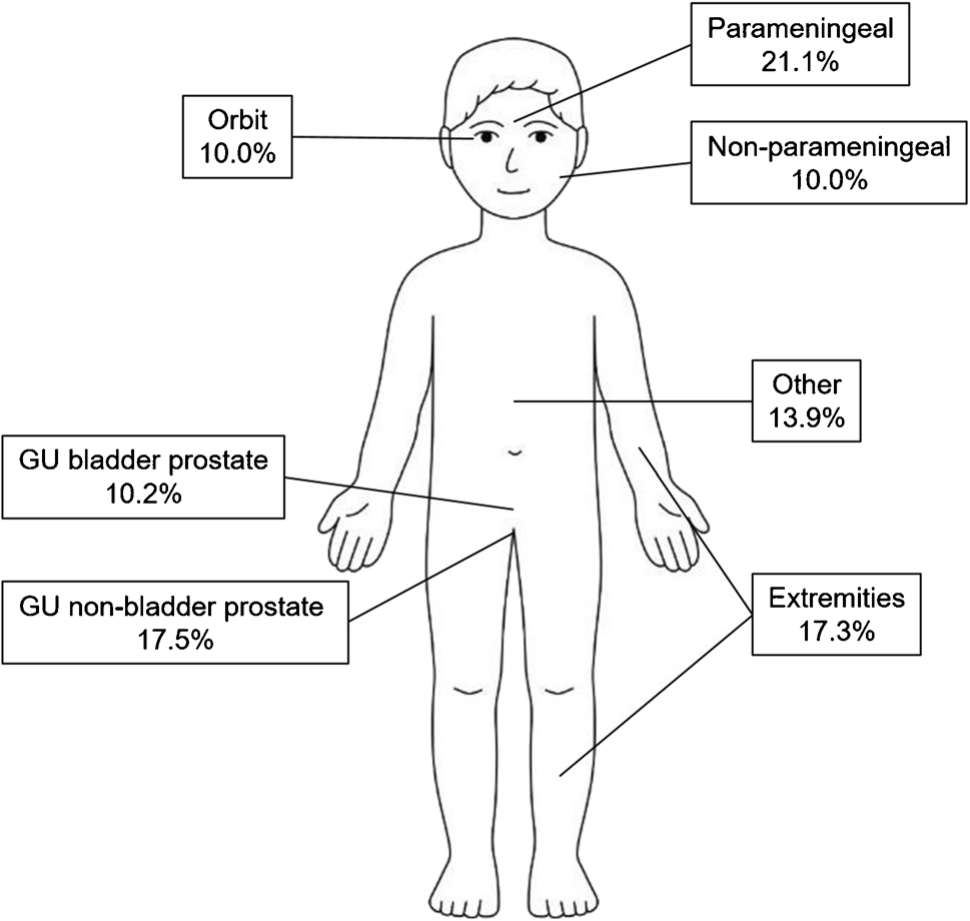
Distribution of rhabdomyosarcoma by primary site, based on data registered in the International Soft Tissue Sarcoma Consortium’s database (*n* = 6,879 patients [2]. Data are shown of 6,809 patients due to missing location in 70 cases). *GU* genitourinary

Rhabdomyosarcoma is a rare soft tissue sarcoma occurring predominantly in the paediatric and adolescent population that can arise anywhere in the human body. Although rare, rhabdomyosarcoma is the most common paediatric soft tissue sarcoma and affects around 400 new patients ages 0–19 years each year across Europe, with an annual incidence of four cases per million in this age range [1]. The median age of presentation is 5 years, with 72–81% of patients being younger than 10 years (Fig. 1) [2]. The incidence is higher in boys compared to girls (ratio: 1.4, 95% confidence interval (CI): 1.2–1.6) [3]. Rhabdomyosarcoma most often occurs in the head and neck, genitourinary (GU) tract and extremities, but can also occur at other sites (Fig. 2; Supplementary Information 1 and 2). Clinical presentation and symptoms can be diverse, as they depend on localisation of the primary tumour mass and the presence of metastases.

The cell of origin of rhabdomyosarcoma is still under debate with proposals including myogenic progenitor cells (given the fact that rhabdomyosarcoma has a myogenic phenotype) [4]. Embryonal rhabdomyosarcoma and alveolar rhabdomyosarcoma are the most important subtypes. Embryonal rhabdomyosarcoma is driven by oncogenic driver mutations like *RAS* and *TP53* mutations, whereas alveolar rhabdomyosarcoma is most often characterised by a fusion protein, resulting from a genomic translocation (mostly *PAX3-FOXO1* or *PAX7-FOXO1*). In most cases, the reason for developing a rhabdomyosarcoma remains unknown; however, there is an increased risk in patients with certain genetic syndromes like neurofibromatosis type 1, Li-Fraumeni syndrome, *DICER1* syndrome, Noonan syndrome, Beckwith Wiedemann syndrome and Costello syndrome [5–10]. Improvement in the management of rhabdomyosarcoma, a chemo- and radiosensitive tumour, has been seen over the past few decades due to the collaborative effort of (inter)nationally cooperative working groups. A combination of systemic chemotherapy and local surgery and/or radiotherapy leads to a 5-year overall survival (OS) of approximately 80% in patients with localised disease, with a range of 50.6% (95% CI: 39.7–60.5) in the very high-risk group to 100% in the low-risk group, and 35% in patients with metastatic disease [11–16].

The rarity of rhabdomyosarcoma makes collaborative studies imperative, which in Europe are governed by the European paediatric Soft tissue sarcoma Study Group (E*p*SSG) and the Cooperative Weichteilsarkom Studiengruppe (CWS), including patients in clinical trials in well over 100 clinical centres. In North America, collaborative rhabdomyosarcoma studies are governed by the Children’s Oncology Group (COG). For high-quality rhabdomyosarcoma imaging, collaborative groups of the E*p*SSG, the CWS and the Oncology Task Force of the European Society of Paediatric Radiology (ESPR) have taken the initiative to develop an international guideline, including technical guidance and standardised reporting forms [17]. Moreover, this collaboration is extended in the International Soft Tissue Sarcoma Database Consortium (INSTRuCT), a collaboration of the COG, CWS, E*p*SSG, International Society of Pediatric Oncology—Malignant Mesenchymal Tumour committee (SIOP-MMT), and the Soft Tissue Sarcoma Committee of the Associazione Italiana di Ematologia e Oncologia Pediatrica (STSC AIEOP) within the Pediatric Cancer Data Commons initiative [18]. The aim of this initiative is to lead international harmonisation in order to optimally and collaboratively acquire data and improve the care and outcome for patients with rhabdomyosarcoma [19].

Imaging plays a crucial role in the entire patient journey: from the clinical suspicion, to the definite diagnosis of rhabdomyosarcoma, the staging of disease for optimal risk stratification, evaluation of response to therapy, planning of local therapy and finally, follow-up and detection of relapse. Treatment of rhabdomyosarcoma requires a specialised multi-disciplinary collaboration in which both paediatric radiologists and nuclear physicians play a key role. In this educational paper, the multi-disciplinary team of our national paediatric oncology centre will take the reader along the disease journey of two patients with rhabdomyosarcoma, where clinical considerations and applications of international imaging guidelines in the paediatric population are explained.

## When to suspect rhabdomyosarcoma

**Table:** 

**Fig. 3:**
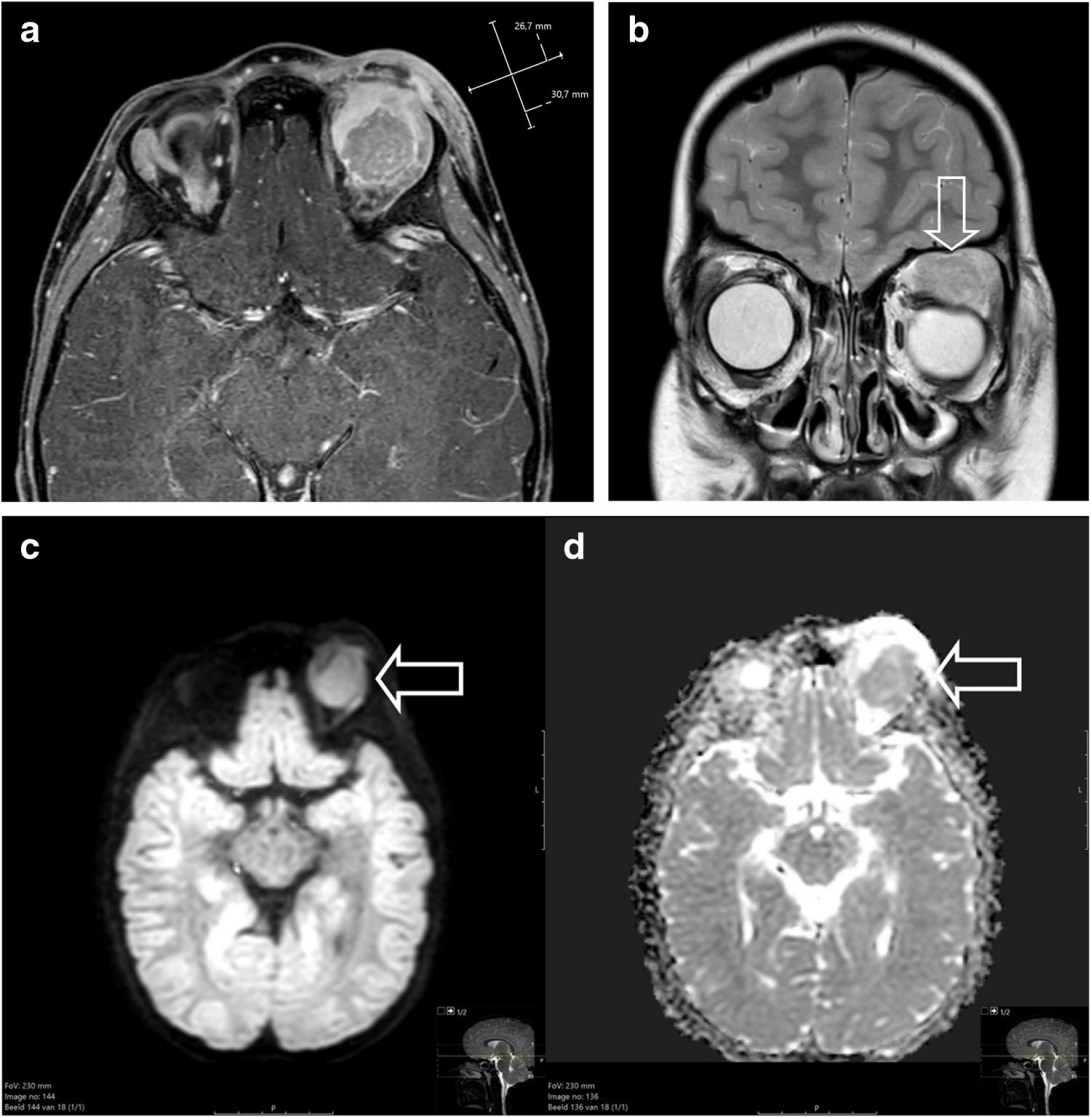
**a**, **b** Magnetic resonance image of a primary orbital tumour in a 6-year-old boy (patient 1) after surgical biopsy and confirmation of rhabdomyosarcoma. **a** Axial T1 after gadolinium administration with fat suppression shows a well-described mass with inhomogeneous enhancement. Post biopsy swelling and subcutaneous and cutaneous oedema are seen. **b** Coronal T2 turbo spin echo image shows a hyperintense signal and compression by the mass of the eye bulb (*arrow*). **c**, **d** Diffusion weighted imaging b-value 800 (**c**) and apparent diffusion coefficient (ADC, **d**) show increased diffusion restriction with hyperintense signal on the b800 and decreased signal on the ADC map (*arrows*)

Patient 1 was a previously healthy 6-year-old boy, who presented to the general practitioner with a persistent swelling of his left eye 4 weeks after minor trauma, accompanied by complaints of progressive visual impairment over the preceding week. On physical examination, a palpable swelling of the left upper eyelid with severe ptosis was evident. There were no signs of infection. Because of the unknown origin of the swelling and progressive visual impairment, the general practitioner referred him to an ophthalmologist. An outside magnetic resonance imaging (MRI) study showed a solid mass in the upper quadrant of the left orbit. Computed tomography of the orbit showed no osseous erosion. A surgical incisional biopsy was performed at the referring hospital.
The patient was referred to a paediatric oncology centre and discussed with a multidisciplinary team. MRI was repeated after the biopsy and showed a mass of 3.1 × 2.7 cm in the upper quadrant with involvement of the surrounding eye muscles (superior and lateral rectus muscles and levator palpebrae superioris muscle) and the lacrimal gland (Fig. 3). No signs of osseous or optic nerve involvement were seen. There was minor compression of the eye bulb. The mass showed enhancement after gadolinium administration and a hyperintense T2 signal. Diffusion-weighted imaging showed increased diffusion restriction. Diffuse swelling and oedema of the eyelid were seen due to the biopsy. No suspicious local lymph nodes were seen. Taken together, clinical and radiological signs were suspicious of an orbital malignancy, possibly a rhabdomyosarcoma. Potential other diagnoses included lymphoma, leukaemia and Langerhans cell histiocytosis.
Histomorphology showed an embryonal rhabdomyosarcoma; as expected with this type of histomorphology, molecular analysis showed no fusion in *PAX-FOXO1.*

**Table:** 

**Fig. 4:**
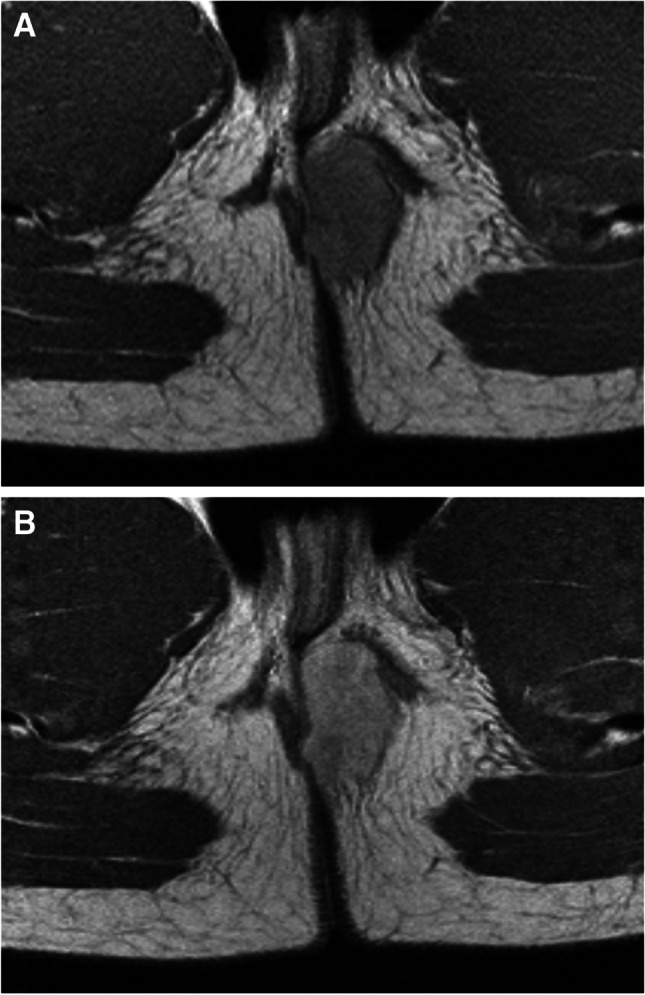
Magnetic resonance image of a primary perianal tumour in a 14-year-old girl (patient 2) with rhabdomyosarcoma. **a** Axial T1 image shows a solid perianal mass with displacement of and invasion into the internal and external anal sphincters. **b** Axial T1 image after administration of gadolinium shows homogeneous enhancement

**Fig. 5:**
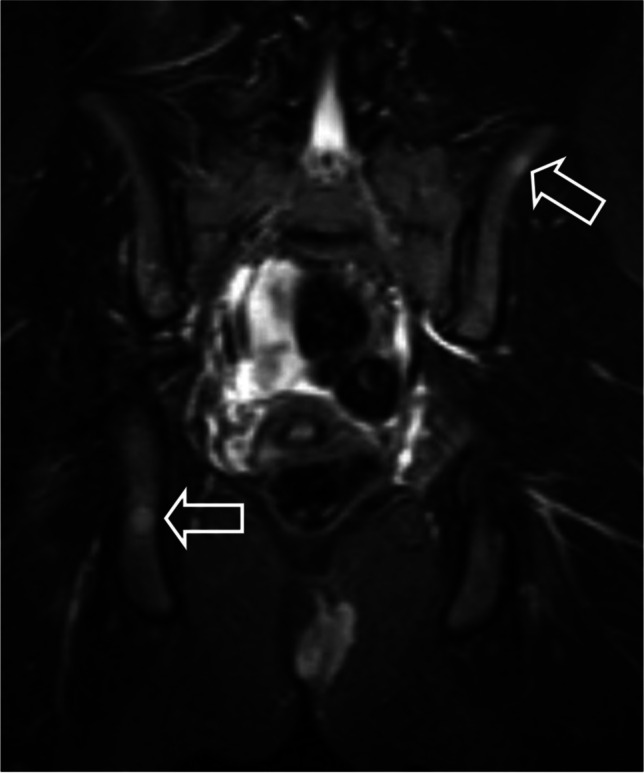
Coronal short tau inversion recovery magnetic resonance image of the pelvis in a 14-year-old girl (patient 2) shows diffuse osseous metastases (*arrows*)

**Fig. 6:**
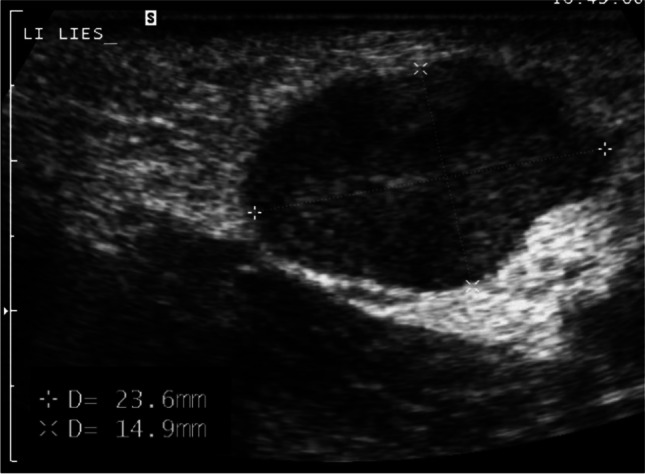
A longitudinal ultrasound image of the left groin a 14-year-old girl (patient 2) shows an enlarged lymph node. Note absence of the normal fatty centre

Patient 2, a girl age 14 years at presentation, felt a small perianal mass and subsequently a slightly enlarged lymph node in her left groin. A month later she visited her general practitioner and was referred to the local hospital. On physical examination, a firm perianal mass measuring 8 × 3 cm and an enlarged firm, ipsilateral inguinal lymph node of 3 × 2 cm were found. There were no signs of infection and neither mass was painful.
An outside MRI, where the groin was not within the field of view, showed a well-demarcated left-sided perianal mass with homogeneous contrast enhancement (Fig. 4). On the coronal short tau inversion recovery (STIR) images, diffuse foci of high signal intensity, in keeping with bone metastases, were visible (Fig. 5). Ultrasound (US) showed a lymph node in the left groin of 1.5 × 2.5 cm, which, due to its short axis of 15 mm, was considered pathologic in line with the RECIST 1.1 criterion. Moreover, on US, a second pathologic enlarged lymph node was seen along the left iliac vein (Fig. 6).
With the findings of a solid soft tissue mass and pathologic enlarged lymph nodes, the differential diagnosis was a rhabdomyosarcoma or an adult-type soft tissue sarcoma. The girl was referred to a paediatric oncology expert centre, where after discussion with the multidisciplinary team the most likely diagnosis was felt to be rhabdomyosarcoma. An incisional biopsy of the primary mass and an excision biopsy of the lymph node in the groin were performed by a paediatric surgeon. Histomorphology showed an alveolar rhabdomyosarcoma, *PAX3-FOXO1* fusion positive, with a tumour-positive lymph node.

Rhabdomyosarcoma is a disease seen mostly in young children that can occur almost anywhere in the body. Rhabdomyosarcoma is locally aggressive and most often presents with a mass or signs caused by the growing mass compromising surrounding tissue. The mass is most often painless. Symptoms thus depend on the location of the tumour and its extent. In general, symptoms mimic common and relatively innocent diseases. The severity or duration of symptoms should alert the physician to the possibility of a malignant tumour. As the presenting symptoms are so diverse and nonspecific, we commonly observe a delay in diagnosis. Rhabdomyosarcoma should be part of the differential diagnosis when confronted with a patient who has a mass of unknown origin with either a prolonged duration and/or a progressive course of the disease. The differential diagnoses of rhabdomyosarcoma are broad and vary depending on imaging features, localisation, size of the tumour and the age of the patient at presentation. Therefore, when confronted with such a patient, it is strongly advised to contact a specialised paediatric oncology centre for diagnostics and further work-up.

Frequently, the first imaging modality in the diagnostic work-up is ultrasonography (US) of the mass. In general, US is easily accessible, can give critical information about the mass and can support an initial differential diagnosis [20]. On US, rhabdomyosarcoma generally presents as a well-defined, slightly hypoechoic inhomogeneous mass that can show increased flow.

If there is suspicion of a soft tissue sarcoma, magnetic resonance imaging (MRI) is the modality of choice to evaluate the tumour characteristics and its relation to surrounding tissue. The field of view of MRI should include the primary tumour, including the locoregional extension of disease, and the locoregional lymph nodes (Table 1). Conventional sequences, such as T1 and T2 with preferably fat-suppressed post-contrast sequences, give morphological information such as localisation and anatomic relationships. Functional sequences, like diffusion-weighted imaging (DWI), provide information on cellular density and diffusion restriction that might suggest a malignant aetiology [21, 22]. Advanced multiparametric imaging can add functional information to the conventional sequences, such as characterisation, volume assessment and response evaluation during or after therapy [23]. The European imaging guideline recommends performing MRI including conventional sequences (T1, T2, post-contrast) in combination with DWI [17]. For the exact protocol, the reader is referred to the aforementioned publication which includes detailed scanner settings for 1.5-tesla (T) and 3.0-T MRI scanners of General Electric, Philips and Siemens. It also provides standard report templates for multiple modalities [17]. In general, because of the heterogeneity of tumour morphology and the influence of necrosis and haemorrhage, soft tissue sarcomas do not have one specific MRI pattern. Usually, they are isointense to muscle on T1 images, hyperintense on T2 images and show avid enhancement after contrast.

**Table 1:** Regional lymph node definition [78]

Region		Definition
Extremities	Upper extremity	Axillary, brachial, epitrochlear, infraclavicular nodes
	Lower extremity	Inguinal, femoral, popliteal nodes
Genito urinary	Bladder/prostate	Pelvic (hypogastric, obturator, iliac, peri-vesical, pelvic, sacral, presacral nodes) (note: para-aortic nodes are distant nodes)
	Cervix	Pelvic (hypogastric, obturator, iliac, peri-vesical, pelvic, sacral, presacral nodes) (note: para-aortic nodes are distant nodes)
	Uterus	Pelvic, retroperitoneal nodes at renal vessels or below
	Paratesticular/gonadal	Ipsilateral pelvic, retroperitoneal nodes at renal vessels or below (inguinal if the scrotum is involved)
	Vagina	Retroperitoneal, pelvic nodes at or below common iliac vessels, inguinal nodes
	Vulva	Inguinal nodes
Head and neck	Head/neck	Ipsilateral parotid, occipital and cervical nodes (all levels). Tumours close to the midline may show bilateral metastases (plural) and retropharyngeal nodes may be involved in parameningeal tumours
	Orbit/eyelid/cheek/external ear/temporal region	Parotid, ipsilateral jugular, pre-auricular, cervical nodes
Trunk	Intrathoracic	Internal mammary, mediastinal nodes
	Retroperitoneum/pelvis	Pelvic, retroperitoneal nodes
	Intra-abdominal	Sub diaphragmatic, intra-abdominal, iliac lymph nodes; according to site
	Abdominal wall	Inguinal, femoral nodes
	Chest wall	Axillary, internal mammary, infraclavicular nodes
Other	Biliary/liver	Porta hepatis nodes
	Perianal, perineal	Inguinal, pelvic nodes; may be bilateral

Nodes outside these regional node definitions (for a given primary site) should be classified as distant metastases

Computed tomography (CT) of the primary tumour should not be performed but can be added for assessment of osseous involvement, especially in the head and neck region, including the anatomically challenging skull base, e.g., suspected bone erosions of orbital primary tumours, which upgrades orbital rhabdomyosarcoma to the unfavourable parameningeal localisation category.

For primary tumour size, 1-dimensional (D) measurements according to Response Evaluation Criteria In Solid Tumours (RECIST) 1.1 are recommended in the European guideline and used for international studies [17, 24]. However, RECIST has not been evaluated in children and not specifically in rhabdomyosarcoma. Historically, both 2-D and 3-D assessments have been used, but neither has shown superiority [25].

### Biopsy

After the first imaging work-up of the primary tumour, an image-guided biopsy should be performed in the same expert centre as the definitive surgery. The radiologist and surgeon with expertise in (paediatric) oncology should collaborate to determine the exact biopsy site which can be resected at the time of the definitive surgery or treated with radiotherapy, to prevent relapse by seeding of tumour cells [26, 27]. The goal of the biopsy is to obtain material for diagnosis, including immunohistochemistry, molecular analyses and (ideally) tumour tissue banking. The pathologist should be notified prior to the biopsy and the biopsy should be sent fresh to the pathology department, without delay.

There are two exceptions with regard to the biopsy procedure in suspected rhabdomyosarcoma; first, the approach of choice for a paratesticular rhabdomyosarcomas is microscopic radical (R0) inguinal orchiectomy; and second, very small tumours can easily be resected with microscopic negative margins without compromising form or function. Pre-surgical biopsy should not be performed in either of these two groups [28].

### Pathology

**Fig. 7:**
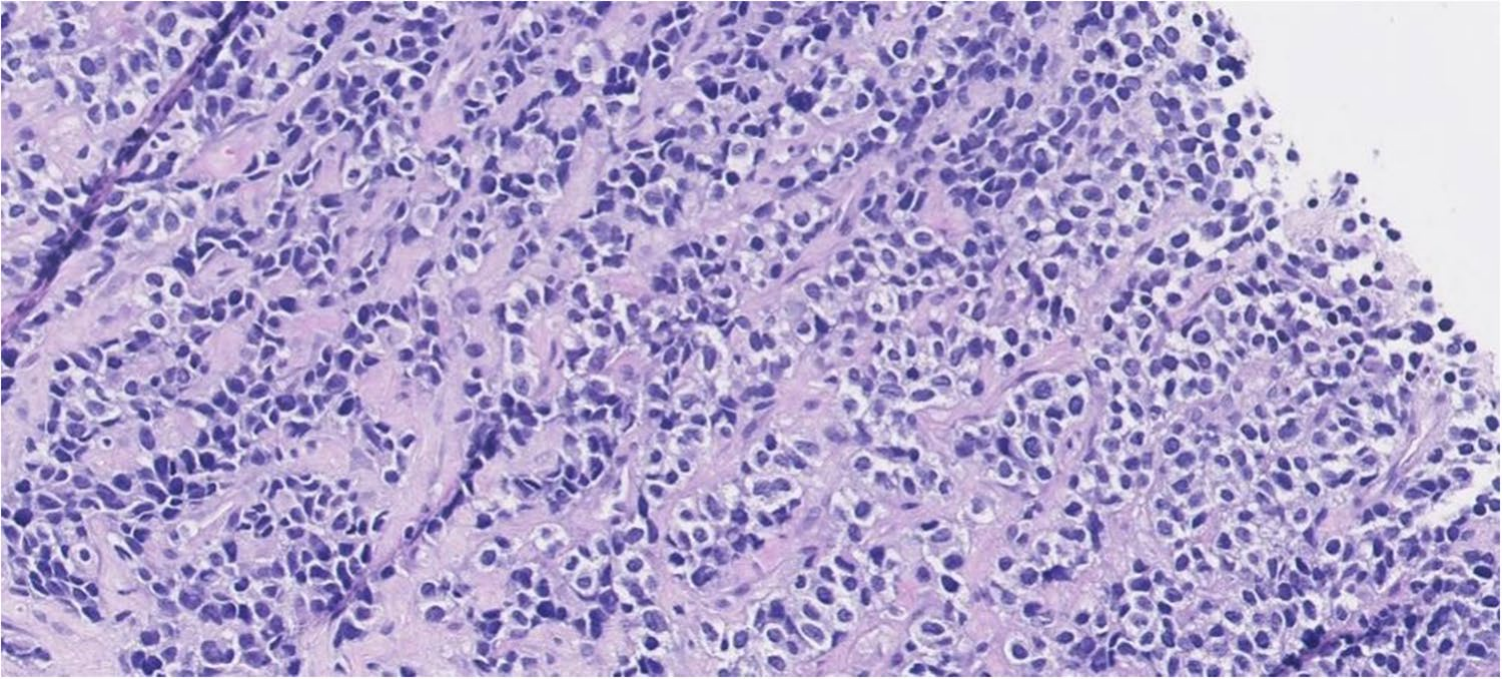
Alveolar rhabdomyosarcoma showing nests of primitive cells with some discohesion in fibrotic stroma (haematoxylin and eosin stain, magnification x20)

**Fig. 8:**
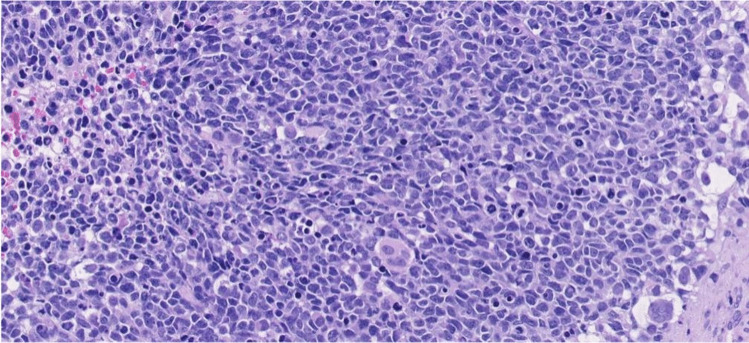
Embryonal rhabdomyosarcoma showing a diffuse proliferation of tumour cells with variable maturation; ranging from primitive cells to rhabdomyoblasts. Note some pleomorphic multinucleated cells (haematoxylin and eosin stain, magnification x20)

In the most recent World Health Organization (WHO) soft tissue tumour classification of 2020, four subtypes of rhabdomyosarcoma are acknowledged: alveolar rhabdomyosarcoma (named after its morphological resemblance to pulmonary alveoli) (Fig. 7), embryonal rhabdomyosarcoma (named after its morphological resemblance to fetal muscle) (Fig. 8), spindle cell/sclerosing rhabdomyosarcoma and pleomorphic rhabdomyosarcoma [29]. Of these subtypes, embryonal rhabdomyosarcoma and alveolar rhabdomyosarcoma are most frequently seen in the paediatric age group. Embryonal rhabdomyosarcoma accounts for 70% of rhabdomyosarcoma in children, with a median age of 6 years. Alveolar rhabdomyosarcoma arises in 25% of children with rhabdomyosarcoma, affecting older children, at a median age of 15 years. Spindle cell/sclerosing rhabdomyosarcoma was first classified as a distinct group in the WHO 2013 classification. This group accounts for 5% of rhabdomyosarcoma in children, affecting children of all ages; in very young children, it is driven by fusion genes (e.g., *VGLL2*) which usually have a good prognosis; in older children, it is caused by a *MYOD1* mutation, which has a dismal prognosis [30, 31]. Pleomorphic rhabdomyosarcoma only appears in adults, has a fundamentally different clinical course and treatment, and therefore is not further discussed in this review.

Morphologically, alveolar rhabdomyosarcoma consists of discohesive nests (classic histology) of tumour cells with round-oval nuclei and minimal cytoplasm (small blue round appearance). Immunohistochemically, there is usually diffuse and strong staining for myogenin (>90%), in addition to positive but more variable staining with desmin and MyoD1. Embryonal rhabdomyosarcoma shows a more heterogeneous histology, consisting of a variably cellular proliferation of tumour cells with a variable degree of maturation, ranging from primitive cells to rhabdomyoblasts. Immunohistochemically, there is variable staining with desmin, MyoD1 and myogenin [32].

**Fig. 9:**
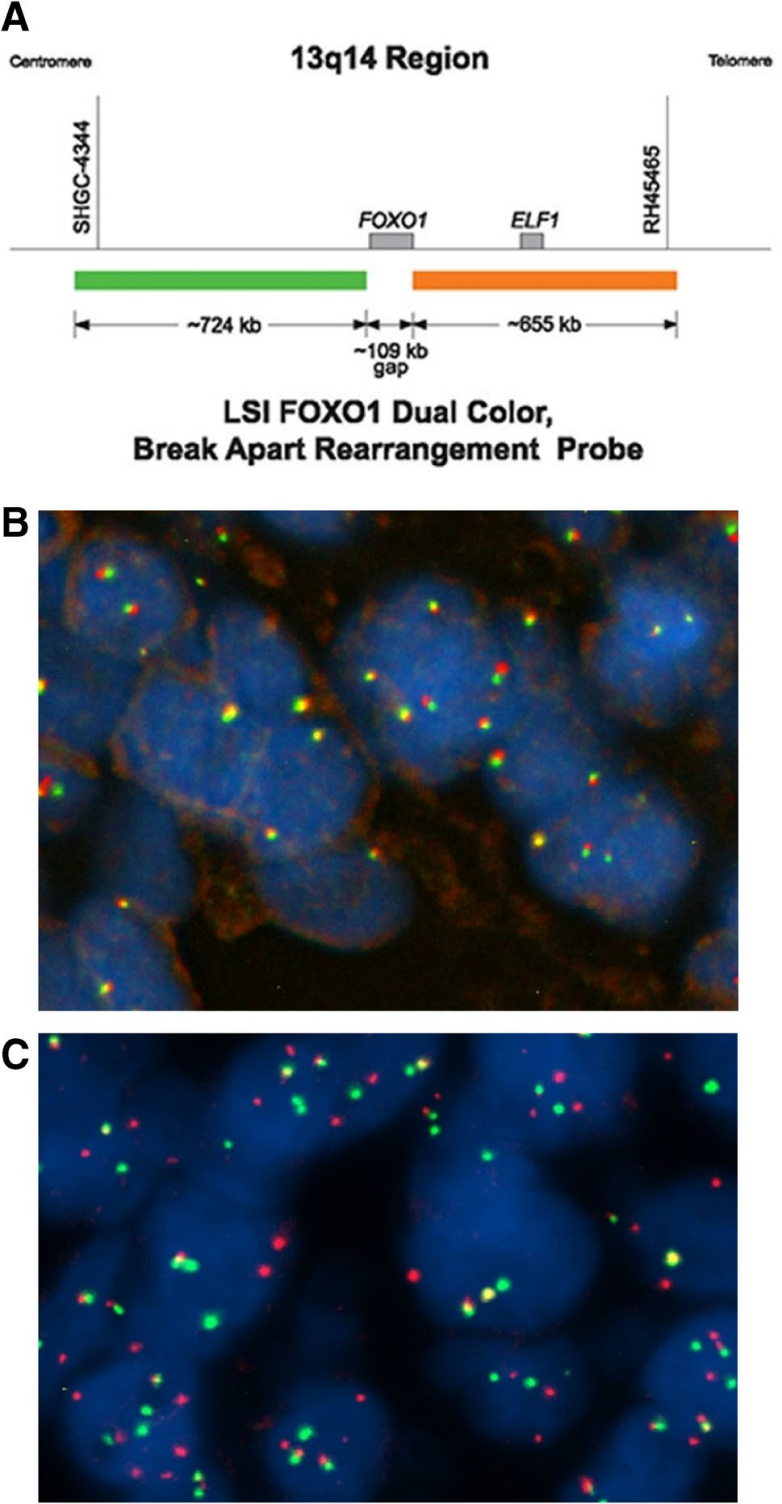
**a** *FOXO1* gene break-apart fluorescence in situ hybridisation (FISH) probe in rhabdomyosarcoma. This probe is an accurate tool to detect the translocations associated with alveolar rhabdomyosarcoma on chromosome 13 (locus 13q14.11) (reprinted with permission from https://www.molecularcatalog.abbott/int/en/Vysis-FOXO1-Break-Apart-FISH-Probe-Kit). **b** *FOXO1*-negative rhabdomyosarcoma. The cells show two fluorescent probes (red and green) located adjacent to each other. **c** *FOXO1*-positive rhabdomyosarcoma, showing separation of the fluorescent probes indicative of a *FOXO1* translocation next to a fused (negative) signal on the normal allele. In this case, additional copies of the *FOXO1* translocated allele were detected

With advancing genetic knowledge, *FOXO1* rearrangements are increasingly recognised (Fig. 9); these genes are found in 70-80% of alveolar rhabdomyosarcoma but not in embryonal rhabdomyosarcoma. In more recent studies, rhabdomyosarcoma is therefore classified as either *FOXO1* fusion positive or fusion negative; patients with fusion positive tumours have a poorer prognosis [14, 15, 33, 34].

**Fig. 10:**
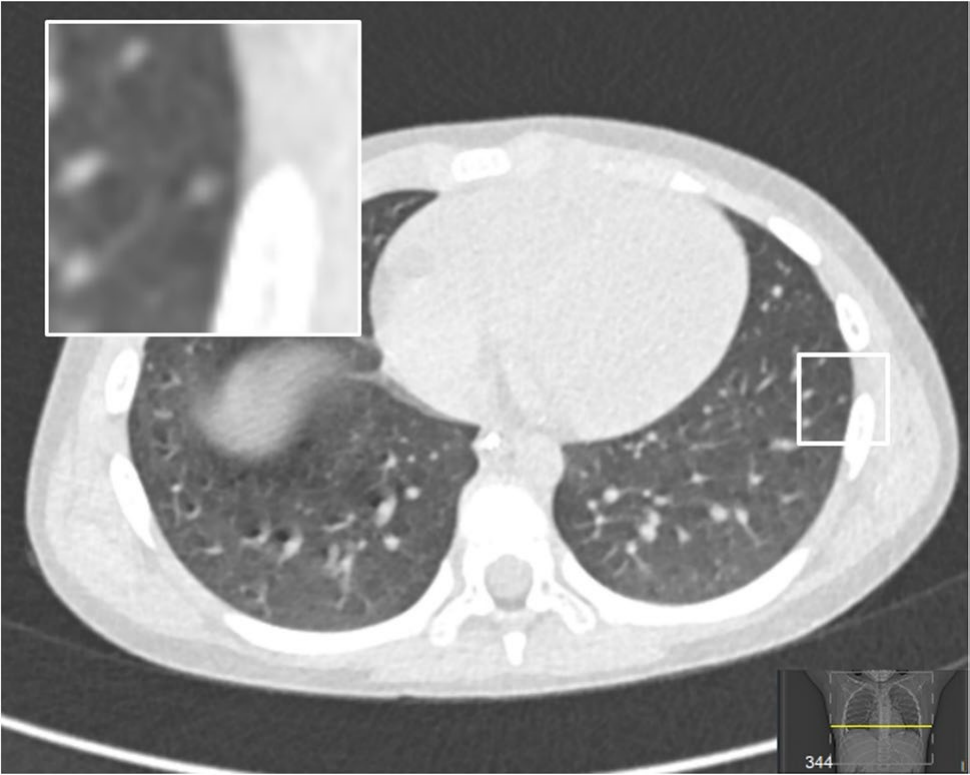
Axial non-contrast enhanced chest computed tomography image of a 6-year-old boy (patient 1) shows a small (<0.5 mm) subpleural nodule (inset). In keeping with the European paediatric Soft tissue Sarcoma study Group guidelines, this nodule was graded as an indeterminate pulmonary nodule, i.e. no pulmonary metastasis

## Staging and risk stratification

**Table:** 

Patient 1
For risk stratification, whole body fluorodeoxyglucose-positron emission tomography FDG-PET/CT was performed, which showed no regional or distant metastases. Chest CT showed a small (<0.5 cm) subpleural lesion (Fig. 10). Because of its size, the nodule was classified as an indeterminate pulmonary nodule.
Bone marrow trephines and punctures showed no bone marrow infiltration.
This patient with embryonal rhabdomyosarcoma, fusion negative, IRS post-surgical stage group III, no nodal involvement, favourable site, size and age, was staged as standard risk, E*p*SSG RMS 2005 treatment group C.

**Table:** 

Patient 2
For risk stratification SPECT/CT was performed, which at that time was standard of care. This showed diffuse bone marrow uptake in keeping with bone metastases. Chest CT showed no pulmonary metastases.
Surgical biopsy of the lymph node in the left groin and bone marrow trephines and punctures showed metastatic disease.
This patient was treated according to the metastatic guidelines, part of the E*p*SSG RMS 2005 protocol.

*CT* computed tomography, *EpSSG* European paediatric Soft tissue Sarcoma study Group, *FDG* fluorodeoxyglucose, *IRS* Intergroup Rhabdomyosarcoma Studies, *PET* positron emission tomography, *RMS* rhabdomyosarcoma, *SPECT* single photon emitted computerised tomography

Once the diagnosis of rhabdomyosarcoma is pathologically confirmed, staging is important for optimal, risk-stratified treatment. Current international risk stratification is based on the patient’s age, tumour site, tumour size, *PAX-FOXO1* fusion status, presence of nodal involvement or metastatic disease and the Intergroup Rhabdomyosarcoma Studies (IRS) post-surgical group staging [14, 15, 35–37]. Based on the MRI, the tumour site (favourable or unfavourable) and the tumour size (≤ or >5 cm) can be assessed, this is further described in the section “Risk stratification groups”. Imaging for staging aims to evaluate the presence of nodal involvement and distant metastases. Rhabdomyosarcoma primarily metastasises to the lungs and bone/bone marrow [38]. However, because rhabdomyosarcoma can metastasise to any site in the body, whole-body imaging is performed for proper staging [17].

The European imaging guideline recommends assessing regional lymph nodes with MRI in combination with fluorodeoxyglucose-positron emission tomography/computed tomography (FDG-PET/CT) or PET/MRI (if available), which not only serves to assess local lymph nodes, but also distant metastases [17]. Additionally, a non-contrast chest CT is standard to evaluate the presence of pulmonary metastatic disease.

### Nodal involvement

The definition of nodal involvement based solely on imaging is a topic of ongoing discussion and collaborative research. In RECIST 1.1, pathological lymph nodes are defined as lymph nodes with a size of  ≥15 mm in short axis. Nodes smaller than 10 mm in short axis are considered non-pathological [24]. The use of RECIST 1.1 criteria for nodal involvement is routinely applied, but not prospectively validated in rhabdomyosarcoma. Importantly, nodes <15 mm may well be positive for tumour. Therefore, FDG-PET positive lymph nodes <15 mm or nodes with abnormal imaging characteristics (abnormal node morphology) should be considered suspicious [24]. Biopsy of suspected positive or ambiguous lymph nodes is strongly advised when possible, as outcome impacts radiotherapy and may impact chemotherapy intensity and duration.

As shown in patient 2, it is essential that the local lymph node stations are included in the field of view. For the retroperitoneum/pelvis, these are the pelvic and retroperitoneal nodes (Table 1). When reporting lymph node basins, it is also important to report on in-transit metastases, i.e. tumour deposits occurring between the primary tumour and proximal draining lymph node basin. In children with distal extremity rhabdomyosarcoma, in-transit metastases were reported to be 50% of all lymph node metastases [39]. FDG-PET/CT improved nodal staging by detecting more regional and in-transit metastases [39]. Popliteal and epitrochlear nodes should be considered as true (distal) regional nodes, rather than in-transit metastases. Biopsy of these nodes is recommended, especially in extremity rhabdomyosarcoma of the distal upper or lower limb.

Surgical node staging should be performed, regardless of imaging characteristics, in all children with a rhabdomyosarcoma of the extremities and all patients over the age of 10 years with a paratesticular rhabdomyosarcoma; in both patient categories, random node picking should be performed of the axillary/inguinal nodes and retroperitoneal nodes, respectively, as the chances of subclinical node involvement are high [28, 39, 40]. Sentinel node procedures are the preferred technique, but when not feasible, random node picking is strongly recommended.

### Metastatic disease

The presence of pulmonary metastases is evaluated in all patients at diagnosis by non-contrast chest CT scan. The European imaging guideline advises a slice thickness of 1.0–1.5 mm and the use of maximum intensity projection to improve sensitivity for detecting pulmonary nodules [17, 41] As shown by Samim et al., small pulmonary nodules (with a mean diameter of 3.2 mm) are a normal finding in 38% of children (95% CI: 26–49%) [42]. Therefore, not every nodule should be categorised as a metastasis. With this in mind, it is important to report pulmonary nodules according to the E*p*SSG crieteria [43]*.* Based on these criteria, three categories of findings are defined as follows: (1) no metastatic disease, defined as no pulmonary lesions present; (2) indeterminate pulmonary lesions, defined as the presence of either: one well-defined nodule measuring 5 mm to 10 mm in diameter or; maximum of four well-defined nodules smaller than 5 mm in diameter; and (3) pulmonary metastases, defined as the presence of either: one or more pulmonary nodules of 10 mm or more in diameter; two or more well-defined nodules of 5 mm to 10 mm in diameter or; five or more well-defined nodules smaller than 5 mm in diameter.

The justification for the definition of indeterminate pulmonary nodules is based on the retrospective evaluation of 316 children with non-metastatic rhabdomyosarcoma who were enrolled on the E*p*SSG RMS 2005 study and equally treated [43]. After a review of the CT scans, 67 patients (21.2%) had indeterminate pulmonary nodules while 249 patients (78.8%) had no pulmonary nodules at diagnosis. After a median follow-up of 75 months, the 5-year OS was 82.0% (95% CI: 69.7–89.6%) for patients with indeterminate pulmonary nodules and 80.8% (95% CI: 75.1–85.3%) for patients without nodules (*P* = 0.76). As the presence of indeterminate pulmonary nodules did not result in different survival, patients with indeterminate pulmonary nodules should be considered and treated as having localised disease. Biopsy of indeterminate lesions is not advised.

To detect distant metastasis, a whole body (head to toe) FDG-PET/CT or PET/MRI is recommended in combination with a non-contrast chest CT. For FDG-PET/CT, European Association of Nuclear Medicine Research Ltd reconstruction is recommended, as this has been standardised by the European Association of Nuclear Medicine (EANM) based on phantom measurements, thereby improving reproducibility between international centres [44–46]. To visually assess FDG uptake by lesions suspected to be malignant, the Deauville criteria are well-known and straightforward [47, 48]. These criteria score the most intense uptake in a site of initial disease according to five categories. Score 1 means no uptake, score 2 the same or less uptake than the mediastinum, score 3 more uptake than the mediastinum but the same or less than the liver, score 4 moderately more uptake than the liver and score 5 markedly higher uptake than the liver.

Bilateral bone marrow punctures and trephines are currently performed in all patients. In the case of parameningeal rhabdomyosarcoma, cerebrospinal fluid collection for cytospin and cell count is the current standard to identify possible central nervous system involvement.

### Risk groups

After complete staging, the following five parameters are used for risk stratification (Table 2):

1. *PAX-FOXO1* fusion status, where a negative fusion status is favourable.
2. The IRS post-surgical grouping system:

- Group I: Describes a tumour that has been removed completely by surgery.
- Group II: Describes a tumour that has been removed with surgery, but cancer cells remain in the body at the edge of the tissue that surrounded the tumour (called a margin), and/or cancer cells are in the regional lymph nodes (lymph nodes near the site of the tumour).
- Group III: Describes a local tumour, which is a tumour that has not spread outside of the area where it started but cannot be completely removed by surgery.
- Group IV: Describes a tumour that has distant metastases. A distant metastasis is a cancer that has spread through the lymph system or blood to another part of the body.

3. The site of the tumour:

- Favourable = Orbit, genitourinary (GU) bladder/prostate, GU non-bladder/prostate (i.e. paratesticular and vagina/uterus), liver-bile duct and head and neck non-parameningeal.
- Unfavourable = All other sites (parameningeal, extremities and “other site”).

4. Nodal stage according to the TNM classification.
5. Size and age:

- Favourable = Tumour size (maximum dimension) ≤5 cm and patient age <10 years).
- Unfavourable = All others (i.e. size >5 cm or age ≥10 years).

**Table 2:** Rhabdomyosarcoma risk stratification

Risk group	Sub-group	*FOXO1* fusion status	Post surgical stage	Site	Node stage	Size and age
Low risk	A	Negative	I	Any	N0	Both favourable
Standard risk	B	Negative	I	Any	N0	One or both unfavourable
	C	Negative	II, III	Favourable	N0	Any
High risk	D	Negative	II, III	Unfavourable	N0	Any
	E	Negative	II, III	Any	N1	Any
	F	Positive	I, II, III	Any	N	Any
Very high risk	G	Positive	II, III	Any	N1	Any
	H	Any	IV	Any	Any	Any

Finally, based on risk stratification, the patient may undergo standardised treatment which, in the majority of cases, is guided by international trials [49]. Depending on regional preferences, treatment is based on either E*p*SSG, CWS or COG study protocols.

## Treatment

**Table:** 

**Fig. 11:**
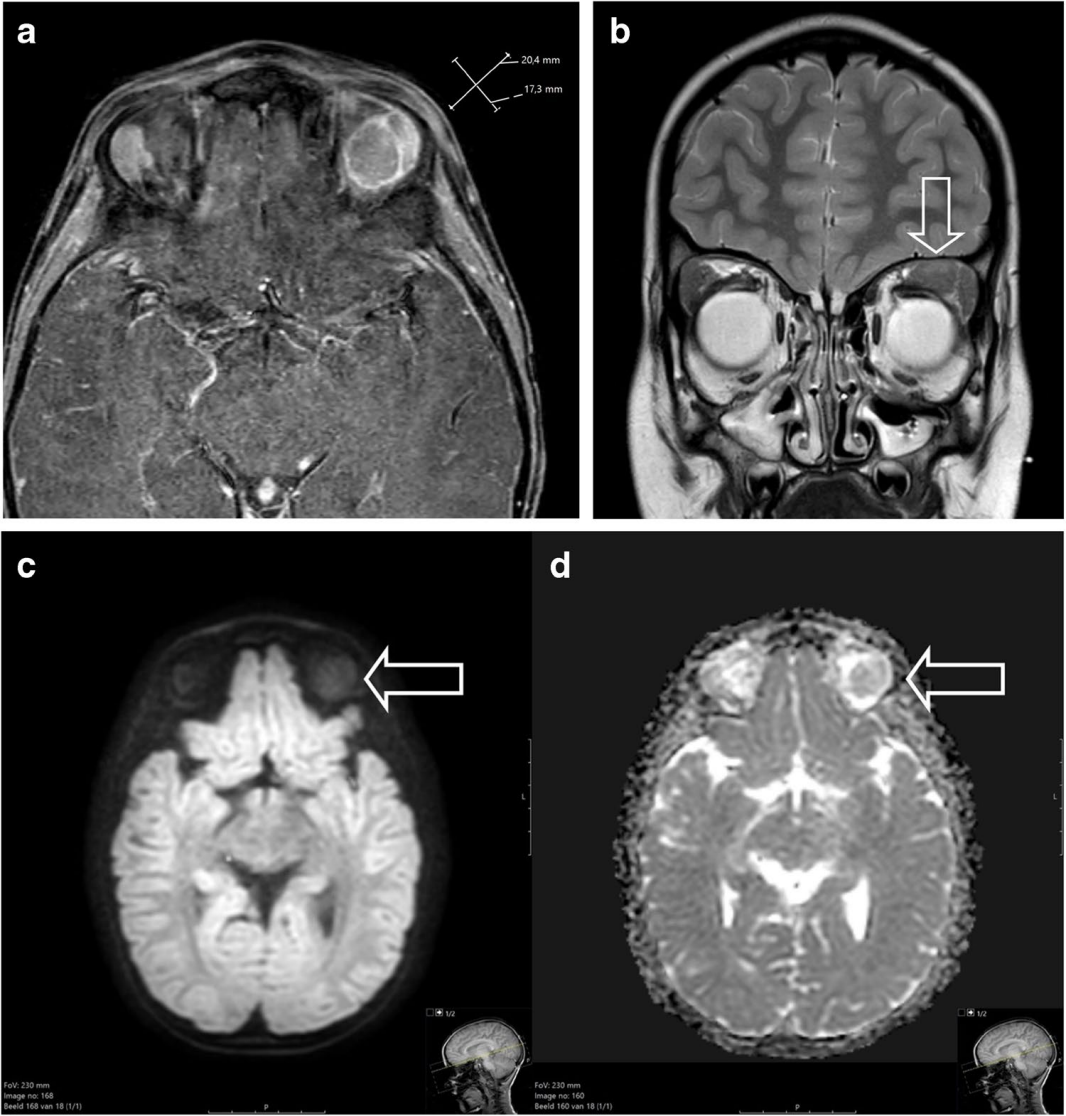
Response assessment magnetic resonance imaging (MRI) of a primary orbital rhabdomyosarcoma in a 6-year-old boy (patient 1) after three cycles of chemotherapy (comparison is made with the baseline MRI study . See also Fig. 3). **a** Axial T1 image after gadolinium administration with fat suppression shows partial remission of the tumour (30–100% volume response) after three months of chemotherapy. **b** Coronal T2 turbo spin echo image shows decreased signal intensity and diminished compression on the globe (*arrow*). The axial diffusion-weighted (b-value 800) (**c**) and axial apparent diffusion coefficient mapping (**d**) images show less restricted diffusion (*arrows*)

Patient 1
The patient was started on chemotherapy which consisted of nine cycles of IVA (ifosfamide, vincristine and actinomycin-D) as defined in the E*p*SSG RMS 2005 protocol. After three cycles, the tumour response was assessed using magnetic resonance imaging. The primary tumour showed volume reduction, a decreased signal intensity on T2 and less restricted diffusion (Fig. 11). Complete surgical resection was not feasible due to the location of the tumour (with involvement of essential eye muscles and the lacrimal gland). As there had been no progressive disease, the planned treatment of chemotherapy and local therapy continued.
Proton therapy was given as local therapy (36 Gy in 1.8 Gy fractions, plus a boost of 9.0 Gy in 1.8 Gy fractions) starting in parallel with chemotherapy cycle five. During this therapy, chemotherapy was continued with ifosfamide and vincristine but without actinomycin-D due to its photosensitising properties.

*EpSSG* European paediatric Soft tissue Sarcoma Group, *RMS* rhabdomyosarcoma

**Table:** 

**Fig. 12:**
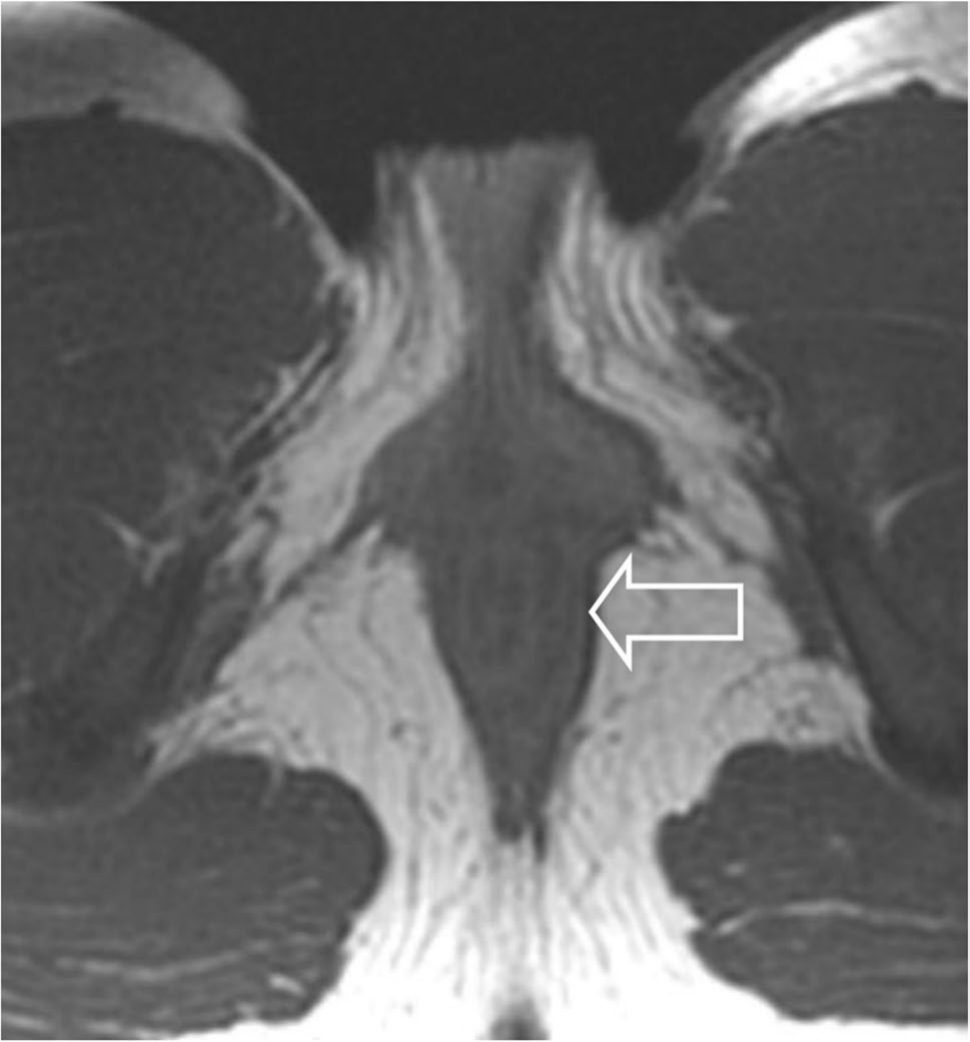
Axial T1 response assessment magnetic resonance image of a 14-year-old girl (patient 2) after two cycles of chemotherapy (due to optimal response after two rather than the standard three cycles) shows minimal residual local disease (*arrow*)

Patient 2
The patient was included in the BERNIE study [50]. Induction therapy consisted of nine cycles of chemotherapy, comprising four cycles of IVADo (ifosfamide, vincristine, actinomycin-D and doxorubicin), followed by five cycles of IVA. Patients were randomised to receive (experimental arm) or not receive (control arm) bevacizumab. Maintenance chemotherapy comprised 12 cycles of low-dose cyclophosphamide and vinorelbine, with or without bevacizumab. The patient was randomised to the standard arm, without bevacizumab.
After three courses, the primary tumour showed a partial response (Fig. 12) while the bone metastases showed complete response. As there was no progressive disease, the planned treatment of chemotherapy and local therapy continued. Local treatment consisted of brachytherapy (55 Gy—44 pulses of 1.25 Gy) of the primary tumour followed by photon radiotherapy of the groin and iliac lymph nodes, with a total dose of 45 Gy.

Therapy of rhabdomyosarcoma always consists of systemic chemotherapy in combination with radiotherapy and/or surgery. Chemotherapy is necessary in all cases, even in the low-risk group, to treat potential micrometastases, which are present in the vast majority of cases, despite normal findings on imaging.

### Chemotherapy

Patients stratified to low-risk groups can be cured with minimal chemotherapy consisting of 22 weeks of vincristine and actinomycin-D (VA) and either no or very low cumulative doses of alkylating agents, thereby decreasing late effects. The mainstay of chemotherapy for the majority of patients with localised disease is combination therapy of nine 3-weekly cycles of IVA (ifosfamide, vincristine and actinomycin-D). 

The E*p*SSG RMS 2005 trial showed that there is no role for the addition of high-dose doxorubicin to standard IVA chemotherapy in patients with high-risk localised disease [12]. The same trial showed that for high-risk patients in clinically complete remission after completion of standard IVA chemotherapy, the addition of six months of low-dose maintenance chemotherapy with vinorelbine and cyclophosphamide significantly improved 5-year OS; 86.5% (95% CI: 80.2–90.9) on maintenance chemotherapy versus 73.7% (95% CI: 65.8–80.1) without (hazard ratio 0.52 (95% CI: 0.32–0.86); *P* = 0.0097).

Patients with node-positive, fusion-positive (E*p*SSG risk group G) and metastatic disease (risk group H) are treated with the most intense regimens and receive four cycles of IVA chemotherapy with doxorubicin (IVADo), followed by five IVA cycles and one year of maintenance chemotherapy [13].

The above summarises the European chemotherapy approach; the most important difference compared to the COG approach is that COG uses cyclophosphamide rather than ifosfamide. Cyclophosphamide and ifosfamide have been shown to be equally effective, but have different adverse effect profiles [51].

### Surgery

The quality of resection at diagnosis is one of the parameters for risk stratification (IRS post-surgical grouping, see above). The aim is complete resection of the primary tumour (R0: microscopic complete resection) without mutilating the patient. In most patients, surgery at diagnosis will therefore be restricted to a diagnostic biopsy (IRS group III). Definitive surgery is considered after four initial cycles of induction chemotherapy to reduce the size and extent of the tumour, allowing for a more conservative surgical approach [27, 52]. If on initial surgical resection, no negative margins are achieved, the tumour is considered to be postsurgical stage IRS group II (microscopic residual, R1) or group III (macroscopic residual, R2).

A pre-treatment re-excision after a primary R1 or R2 resection is feasible when this can be done with preservation of form and function. If residual tumour remains after induction chemotherapy, local therapy should be discussed by the multidisciplinary team, considering delayed primary excision and/or radiotherapy. The goal of local therapy is achieving maximum local control with minimum harm to the patient.

### Radiotherapy

Radiotherapy of the site of the primary tumour is indicated for most patients, particularly those in the high-risk and very high-risk groups and the majority of standard-risk patients [53, 54]. There are a few exceptions: localised fusion-negative rhabdomyosarcoma with initial R0 resection (IRS Group I), localised fusion-negative rhabdomyosarcoma of the vagina achieving complete remission with induction chemotherapy, and a highly selected group of patients with IRS Group II/ III fusion-negative rhabdomyosarcoma, arising at a favourable site, where secondary surgery achieves an R0 resection (e.g. paratesticular, uterus).

In addition, radiotherapy should be delivered to all regional nodal sites involved at the time of presentation, irrespective of response to induction chemotherapy and/or any additional surgical resection. Lymph node dissections are strongly discouraged.

Patients with metastatic disease should receive radiotherapy to their primary tumour, involved regional nodes and all sites of metastatic disease that can feasibly be treated, especially remaining active metastases after induction chemotherapy [54].

For very young patients, especially those below 1 year of age, decisions on radiotherapy should be discussed with (inter)national expert teams to assess the survival benefit against potential adverse effects of radiotherapy. Depending on the field of radiotherapy, late adverse effects can include cardiovascular disease, muscular hypoplasia, growth arrest/retardation (leading to deformity) and secondary malignancies [55]. For optimal planning of radiotherapy, imaging of the primary tumour and its metastatic sites, at diagnosis and at response assessment, is essential.

Historically, the COG was more rigorous in its use of radiotherapy, however, more recently, the radiotherapy approaches on both sides of the Atlantic have become much more alike, resulting in an increase in the use of radiotherapy in Europe.

### Brachytherapy

In the head and neck area, a microscopic radical delayed resection is often hard to achieve without extensive mutilation and, therefore, local radiotherapy is often preferred. However, external beam radiotherapy (EBRT) is known to impair growth and function in young children. To minimise late adverse events, the AMORE technique, an acronym for, Ablative surgery, MOulage brachytherapy and surgical REconstruction, was developed [56]. The advantage of brachytherapy is a highly conformal dose delivery to the tumour bed with rapid dose fall-off beyond the treatment volume, therefore sparing the surrounding tissue.

AMORE has shown to result in similar OS and a reduction of late adverse effects when compared to EBRT [56–59]. In addition, for patients with relapsed disease, AMORE may offer an effective salvage option [60]. Finally, for the bladder/prostate region in younger boys and the female genital tract, combined surgery and brachytherapy approaches offer adequate survival with better functional and aesthetic outcomes [61, 62]. It should be noted that (combined) brachytherapy techniques can only be executed in a few international highly-specialised centres of expertise.

## Response assessment and follow up

**Table:** 

**Fig. 13:**
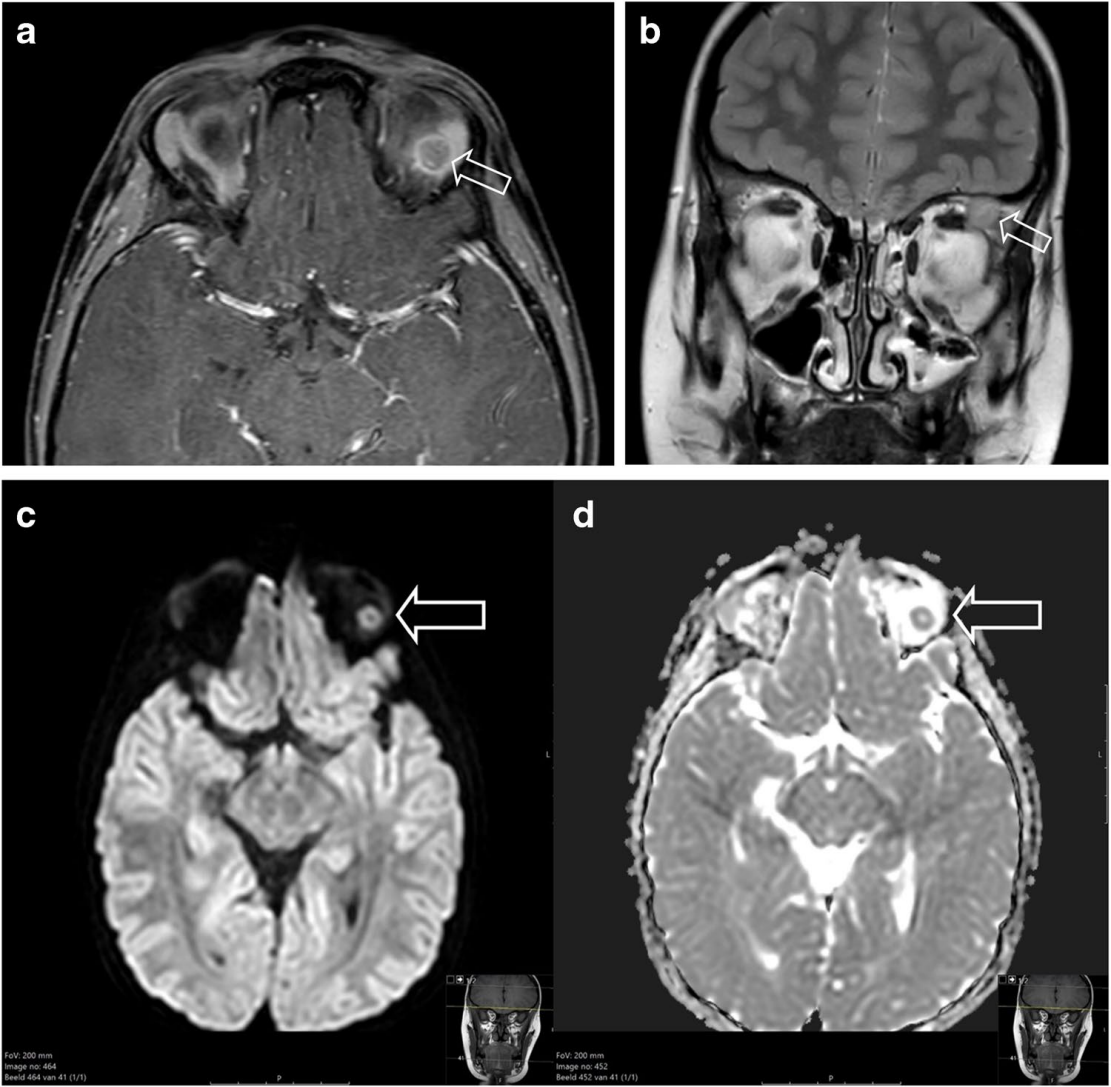
Follow-up magnetic resonance imaging of a primary orbital rhabdomyosarcoma in a 6-year-old boy (patient 1), 1 year after initial diagnosis. Axial T1 (**a**) and coronal T2 turbo spin echo (**b**) images following gadolinium show tumour recurrence (*arrows*). Axial diffusion weighted, (b-value 800) (**c**) and axial apparent diffusion coefficient mapping map (**d**), show a focus of diffusion restriction in keeping with recurrence (*arrows*)

Patient 1
After completion of therapy, further volume reduction of the residual tumour was evident on MRI. An increase in soft tissue oedema was seen due to recent proton therapy. During follow-up, the residual tumour remained stable.
Seven months after the end of treatment, the tumour showed progression on follow-up imaging. MRI showed tumour growth with diffusion restriction (Fig. 13). No signs of intracranial extension were seen.
Due to suspicion of relapse, a FDG-PET/CT was performed, which showed no distant metastases. A biopsy was performed that showed embryonal rhabdomyosarcoma cells.
Relapse therapy was started with VIT chemotherapy (vincristine, irinotecan and temozolomide) [63]. After two courses, the tumour showed stable volume and a loss of diffusion restriction.
The patient was discussed by a multidisciplinary team and it was decided that the tumour residue was resectable, making the patient eligible for salvage AMORE procedure including exenteration of the orbit followed by brachytherapy.
Follow-up MRIs have been performed every 3 months and there are no signs of relapse to date.

**Table:** 

**Fig. 14:**
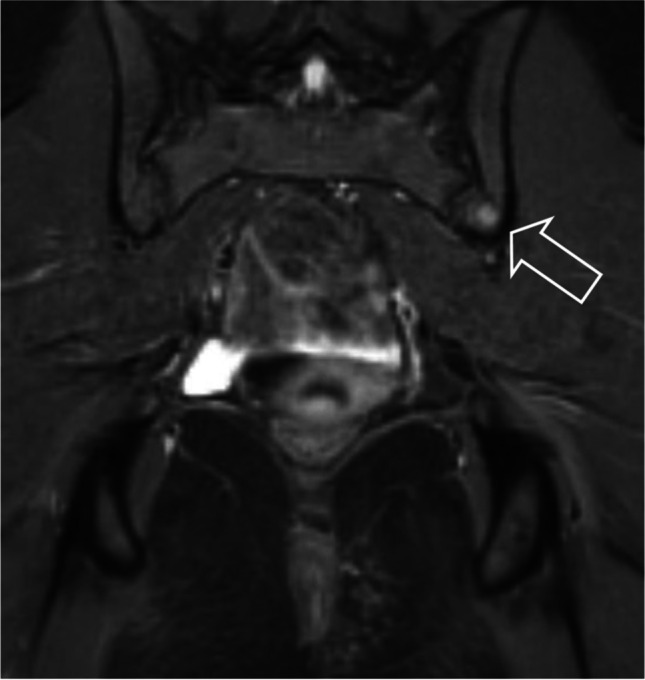
Follow-up coronal T2 magnetic resonance image of the pelvis in a 14-year-old girl (patient 2), 17 months after completion of chemotherapy, shows a small area of high signal intensity (*arrow*)

**Fig. 15:**
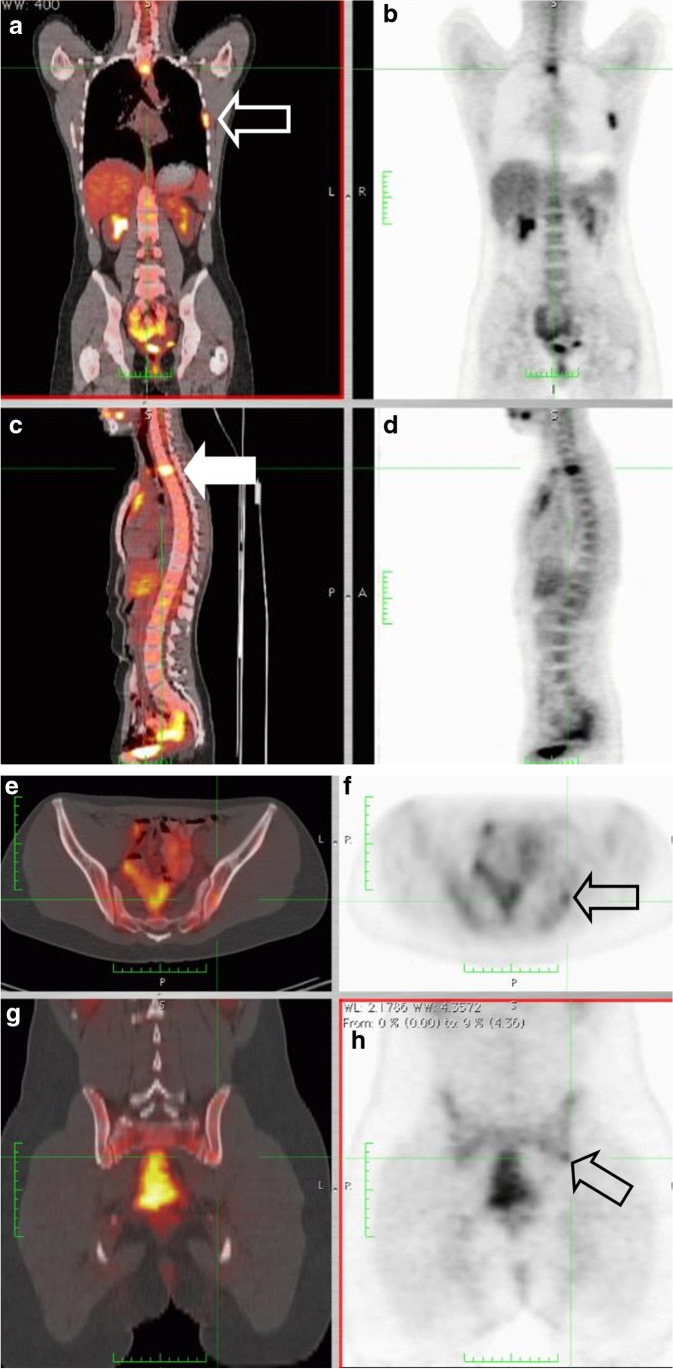
**a**–**h** Coronal (**a**, **b**, **g**, **h**), sagittal (**c**, **d**) and axial (**e**, **f**) fluorodeoxyglucose-positron emission-tomography-computed tomography images in 15-year-old girl (patient 2) approximately 17 months after completion of chemotherapy show multiple metastases with increased tracer uptake of the left 3rd rib (**a**, **b**) (*arrow* in **a**), the 12th thoracic vertebral body (**c**, **d**) (*arrow* in **c**) and osseous metastases in the pelvis (**e**–**h**) (*arrows* in **f** and **h**)

**Fig. 16:**
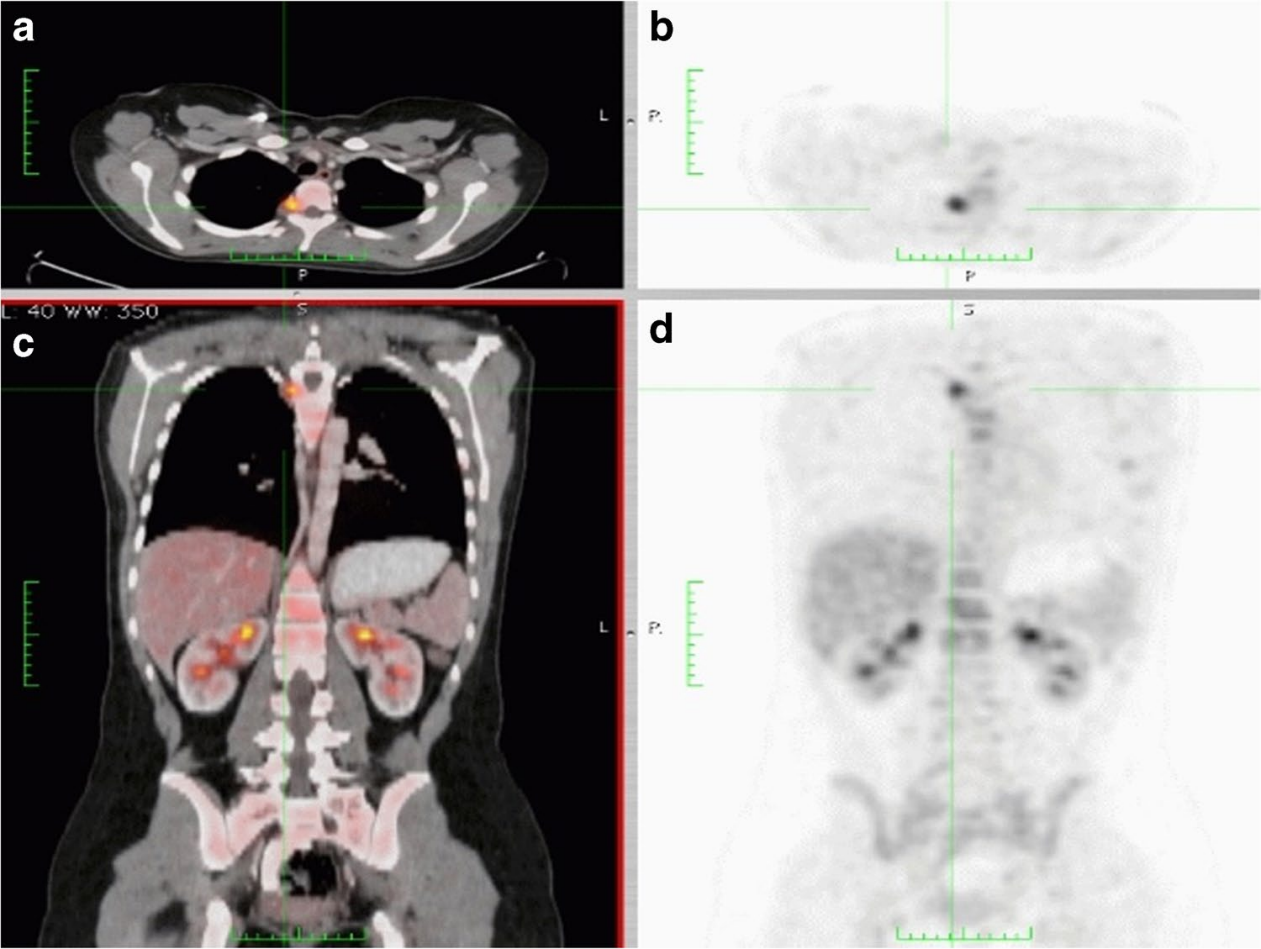
Axial (**a**, **b**) and coronal (**c**, **d**) fluorodeoxyglucose-positron emission tomograhy-computed tomography scans of a 15-year-old girl (patient 2) just before completion of treatment of recurrent rhabdomyosarcoma show new osseous metastases in the spine and pelvis

**Fig. 17:**
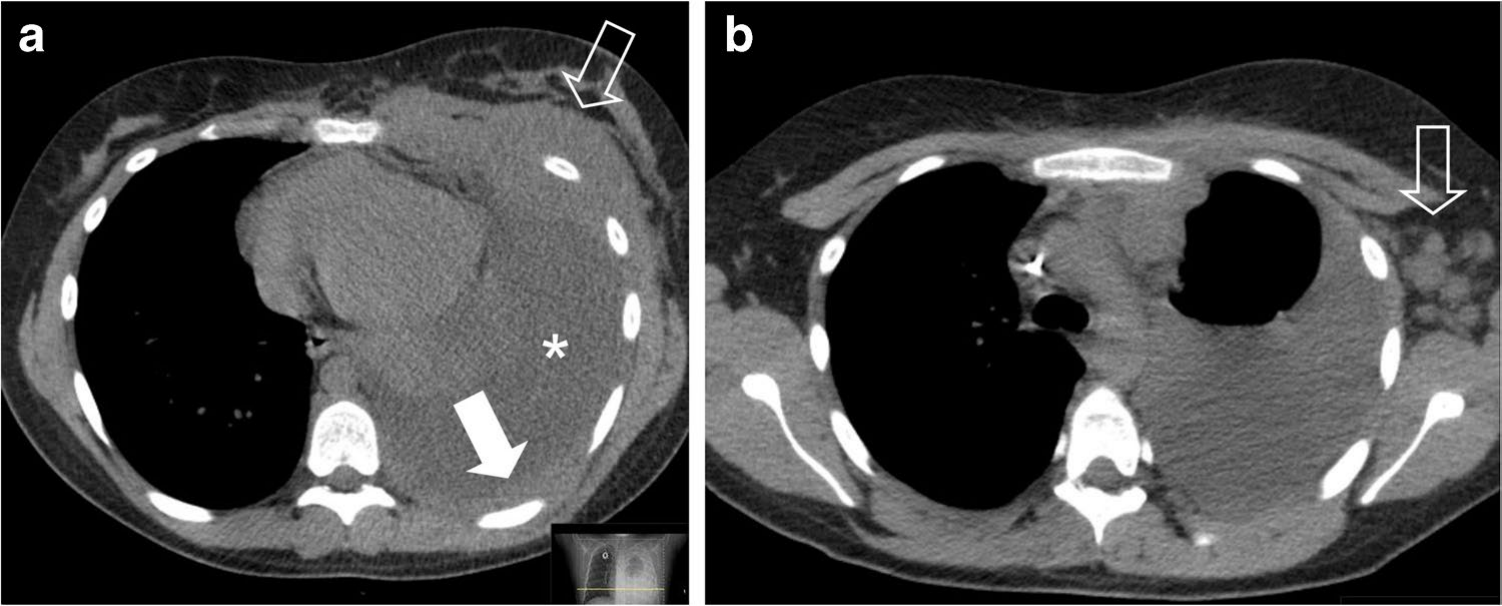
**a**, **b** Axial non-contrast enhanced chest computed tomography scans of a 15-year-old girl (patient 2) with rhabdomyosarcoma show (**a**) a large soft-tissue mass surrounding multiple ribs (*open arrow*) with extension into the chest cavity (*solid arrow*) leading to a malignant pleural effusion (*asterisk*) and (**b**) multiple axillary lymph node metastases (*arrow*)

Patient 2
Almost 17 months after achieving complete remission, the patient presented with a new pelvic skeletal metastasis on MRI (Fig. 14). FDG-PET-CT showed multiple skeletal metastases (Fig. 15). This recurrence indicated a very poor prognosis, nevertheless the patient and her parents opted for potentially curative second-line chemotherapy. This treatment was based on the COG protocol D9802 and consisted of nine courses of a combination of irinotecan and vincristine alternating with vincristine, actinomycin-D and cyclophosphamide (VAC).
Initially, the patient again reached complete response on imaging. However, just before the end of treatment, a second recurrence was diagnosed (Fig. 16). At that point, no other curative options were available. In the months following recurrence, rapid growth of the metastases was seen (Fig. 17).
Thirty-one months after the initial diagnosis, the patient succumbed to her disease.

AMORE Ablative surgery, MOulage brachytherapy and surgical REconstruction, *COG* Children's Oncology Group, *CT* computerised tomography, *FDG* fluorodeoxyglucose, *MRI* magnetic resonance imaging, *PET* positron emission tomography

During treatment, imaging of the primary tumour and, if present, metastatic sites, is performed to assess tumour response to chemotherapy. The response can either be assessed using RECIST 1.1 (mandatory in pharmaceutical driven studies) or volumetric (Table 3) [24]. Until recently, volumetric response was seen as an early surrogate marker of response and inherent efficacy of chemotherapy, however, recent studies have cast doubt on this assumption. Therefore, in current studies, size response is not used as an endpoint. A recent systematic review by van Ewijk et al. included six studies, consisting of a total of 2,010 patients and assessed the implications of tumour response in relation to event-free survival and OS [64]. Based on their review the authors concluded that ‘*Early progressive disease is associated with poorer survival compared to patients with non-progressive disease, being either stable disease, partial, or complete response. However, for the vast majority of patients with nonprogressive disease, we found no evidence that the degree of response is prognostic for survival. Therefore, the value of early tumour size response as a prognostic marker, and its translation into treatment modifications on an individual patient or trial level should be reconsidered*’ [64]. This means that currently, the degree of volume response does not guide treatment, with the exception of patients with progressive disease, which is shown to correlate with a poor prognosis [65, 66]. In RECIST 1.1, as well as the detection of new lesions, a 1-D increase of 20% is also classified as progressive disease, which correlates to a 3-D volumetric increase of 73%.

**Table 3:** Definitions of primary tumour response

	1-dimensional assessment (RECIST 1.1)	3-dimensional volumetric assessment
CR: complete remission	100% decrease	100% decrease
PR: partial remission	≥30%, but <100% decrease	≥66%, but <100% decrease
SD: stable disease	Neither PR nor PD	Neither PR nor PD
PD: progressive disease	≥20% increase	≥73% increase

Currently, no functional imaging studies are standard of care for response assessment in rhabdomyosarcoma. Therefore, there is an imminent need for new surrogate markers; this perspective will be discussed in the next section.

After treatment, up to one-third of patients with initial non-metastatic disease will show tumour recurrence, most often a locoregional relapse [12, 67]. Recurrence rates for patients with initial metastatic disease are up to 70% [14]. Distant metastatic recurrences occur more often in patients with initial metastatic disease. After recurrence, OS in patients with initial non-metastatic disease is approximately 37% [68]. For patients with initial metastatic disease, survival rate after recurrence is very low.

All treated patients undergo strict and intensive surveillance with imaging of the primary tumour site, chest radiography and a general consultation with the paediatric oncologist. The assumption is that surveillance imaging leads to earlier detection of recurrence and therefore to improved outcomes after recurrence. In recent years, this hypothesis has been questioned [69–71]. Several studies in adults showed no benefit of post-therapy surveillance on survival [72–74]. Furthermore, this surveillance also generates feelings of anxiety and stress, especially surrounding the surveillance scan and the period between the scan and the result of the scan, this feeling is known as ‘scanxiety’ [74–77].

Vaarwerk et al. retrospectively assessed the value of surveillance imaging in children with initially localised rhabdomyosarcoma [76]. A total of 199 children with relapsed disease were included in the study; in 121 patients (60.8%), relapses were detected as a result of presentation with clinical symptoms related to recurrence in between surveillance scans. Twenty-two cases (11.1%) were detected by surveillance imaging although clinical symptoms were present at the time, and in 56 cases (28.1%), recurrence was diagnosed solely on surveillance imaging findings. After combining the last two groups, the 3-year OS was 46% (95% CI: 37–55%) for clinically symptomatic patients and 50% (95% CI: 38–61%) for surveillance imaging patients (*P* = 0.7). Similar findings were reported by Fetzko et al., using data from 127 patients treated in five tertiary North American paediatric cancer centres. These authors found no significant difference in the 4-year OS after relapse between patients with relapse detected by imaging (28%, 95% CI: 14–40%) and patients with relapse detected because of clinical symptoms (21%, 95% CI: 11–31%, *p* = 0.14) [78]. Using data from 43 children treated at Texas Children’s Hospital, Lin et al. also found no difference in OS between patients in whom progression or relapse was diagnosed based on imaging versus clinical evaluation (3-year survival 20% vs. 11%, *P* = 0.38) [79].

The current European imaging guidelines advise that surveillance imaging should be performed three times a year in the first 2 years after the end of therapy, reducing the period of surveillance of the primary tumour from 5 to 2 years.

However, taking into account the limited evidence for a beneficial effect of surveillance imaging on survival, we would like to echo the words of McHugh and Roebuck ‘*To resolve the question of the value of ongoing imaging surveillance, patients should be randomized to either of two arms once therapy is complete; one with routine surveillance imaging, the other without surveillance imaging (relying solely on clinical follow-up)*’ [69].

## Future perspectives

The E*p*SSG launched their new overarching study for children and adults with newly diagnosed and relapsed rhabdomyosarcoma (FaR-RMS) in September 2020 [80]. This prospective multi-arm, multi-stage study aims to recruit 1,672 patients in 24 countries across Europe, Israel, Australia, New Zealand and Canada. It is designed to answer nine questions for randomised trials on chemotherapy and radiotherapy, and includes studies on the development of imaging and biology biomarkers. By virtue of its multi-arm multi-stage design, it is possible to introduce new treatment arms, evaluating the impact of new agent regimens in both newly diagnosed and relapsed rhabdomyosarcoma, while the overarching study is still running.

In current rhabdomyosarcoma trials, survival is the only available valid, but late, endpoint. Consequently, it often takes between 7 to 10 years to answer just a few important clinical questions [12]. This highlights the urgent need to develop early biomarkers to enable (1) at the clinical trial level, early evaluation of the efficacy of new treatment regimens, to allow for early selection of effective regimens; and (2) at the individual patient level, prediction of treatment efficacy to allow for timely switch to second-line therapy in case standard treatment appears to be ineffective. Therefore, within FaR-RMS, there are two major radiology and nuclear imaging-driven and led sub-studies; an FDG-PET/CT response study and a DWI response study. Each study aims to evaluate the technique’s potential as an early prognostic biomarker for outcome. As the success of multicentre imaging studies depends on data homogeneity, the E*p*SSG, CWS and European Society of Paediatric Radiology have collaboratively published a detailed imaging protocol for rhabdomyosarcoma, including guidelines for reporting. [17].

Another prerequisite for a successful multinational multicentre study is the availability of a platform to share data. This platform should not only adhere to all standards and regulations, e.g., Good Clinical Research Practice (GCP) [81] and the General Data Protection Regulation (GDPR) [82], but should also be easy to use. The ease of use is especially important when dealing with rare diseases, as many centres will only encounter a handful of patients annually. A suitable solution for this is the ‘Quality and Excellence in Radiotherapy and Imaging for Children and Adolescents with Cancer across Europe in Clinical Trials’ (QUARTET) project, which is driven by the European Society for Paediatric Oncology (SIOPE) [83, 84]. QUARTET is embedded in the infrastructure of the European Organisation for Research and Treatment of Cancer (EORTC), ensuring a stable and practice-proven platform.

Currently, the INSTRuCT database holds clinical information on 6,969 rhabdomyosarcoma patients. This makes it possible to analyse subsets of patient groups that, given their rarity, cannot be studied within the individual international collaborative trial databases [32, 53, 85, 86].

The last step to be taken is to implement the FAIR principle (Findability, Accessibility, Interoperability and Reuse of digital assets) in (paediatric) oncology studies [87]. It is especially important in the field of artificial intelligence (AI) where large, well-annotated datasets are essential. The use of a platform like QUARTET combined with the FAIR policy which, to date have been largely underutilised in paediatric radiology, will support the use of AI in paediatric radiology [88].

## Conclusion

With this narrative review, we hope to have provided the reader with an overview of the current status of imaging and treatment of rhabdomyosarcoma in children and to have given them an insight into a bright future where (paediatric) radiologists and nuclear physicians play a leading and vital role in prospective studies.

## Supplementary Information

### Supplementary Information 1

**Table:** Distribution of primary sites for rhabdomyosarcoma based on data registered in the INSTRuCT database (*n*= 6,809 patients) [2]

Primary site		Number of patients	Percentage
Extremity		**1,178**	**17.3%** ^**a**^
	Foot	99	8.4%^b^
	Forearm	150	12.7%
	Hand	97	8.2%
	Knee	5	0.4%
	Lower leg	159	13.5%
	Other extremity	29	2.5%
	Pelvis	370	31.4%
	Shoulder	44	3.7%
	Thigh	180	15.3%
	Upper arm	45	3.8%
Genitourinary (GU) bladder/prostate		**696**	**10.2%**
	Bladder	389	55.9%
	Bladder/prostate	93	13.4%
	Prostate	214	30.7%
GU non-bladder/prostate		**1,196**	**17.5%**
	Cervix	20	1.7%
	Kidney	9	0.8%
	Other GU non-bladder/prostate	25	2.1%
	Ovary	9	0.8%
	Paratesticular	869	72.7%
	Uterus	69	5.8%
	Vagina	167	14.0%
	Vulva	28	2.3%
Parameningeal		**1,438**	**21.1%**
	Brain	3	0.2%
	Hypopharynx	2	0.1%
	Infratemporal fossa/pterygopalatine	118	8.2%
	Infratemporal fossa/pterygopalatine and parapharyngeal area	207	14.4%
	Middle ear	151	10.5%
	Nasal cavity	29	2.0%
	Nasal cavity and paranasal sinuses	204	14.2%
	Nasopharynx	317	22.0%
	Other parameningeal	218	15.2%
	Paranasal sinuses	137	9.5%
	Parapharyngeal area	52	3.6%
Non-parameningeal head and neck		**678**	**10.0%**
	Cheek	130	19.2%
	Larynx	17	2.5%
	Neck	71	10.5%
	Oral cavity	75	11.1%
	Oropharynx	59	8.7%
	Other face	98	14.5%
	Other head and neck	147	21.7%
	Parotid	59	8.7%
	Scalp	22	3.2%
Orbit		**679**	**10.0%**
	Eyelid	58	8.5%
	Orbit	621	91.5%
Other site		**944**	**13.9%**
	Abdomen	170	18.0%
	Anal/perianal	54	5.7%
	Buttock	83	8.8%
	Liver/biliary tract	85	9.0%
	Other	8	0.8%
	Paraspinal	32	3.4%
	Perineum	73	7.7%
	Retroperitoneum	158	16.7%
	Thorax	141	14.9%
	Trunk	140	14.8%

Values in bold indicate sums of the values

^a^Percentage of all patients with known anatomic location of the primary tumour in the International Soft Tissue Sarcoma Consortium database

^b^Percentage of patients within one of the seven main anatomic locations

### Supplementary Information 2

**Table:** Definition of sites of origin [78]

Eyelid	This site is sometimes erroneously designated as “eye”. Although there may occasionally be a case arising from the conjunctiva of the eye, the globe itself is not a primary site. The eyelid is much less frequent than the orbit itself
Orbit	This refers to the bony cavity, which contains the globe, nerve and vessels and the extra-ocular muscles
Parameningeal	Tumour in this site will only rarely invade the bony walls and extend into the adjacent sinuses. This is why this tumour which is clearly adjacent to the skull base and its meninges is not by its natural history appropriate to include in the parameningeal sites, unless there is invasion of bone at the base of the skull
Middle ear	This refers to a primary that begins medial to the tympanic membrane. This tumour is often advanced at presentation and because of extension laterally may present with a mass in front of or under the ear suggesting a parotid origin. It may also extend through the tympanic membrane and appear to be arising in the ear canal. When there is doubt about the site of origin, the “middle ear” designation should be picked as it implies the more aggressive therapy required of parameningeal sites
Nasal cavity and paranasal sinuses	The three paranasal sinuses are the maxillary sinuses, the ethmoid sinuses and the sphenoid sinus. These surround the nasal cavity, and a primary in one will frequently extend to another. It can be difficult to determine the exact site of origin, but the choice is academic as the treatment is not affected. The site designation will have a bearing on the design of radiotherapy portals. Tumour arising in the maxillary or the ethmoid sinuses may invade the orbit. This is much more likely than a primary in the orbit invading one of the sinuses. When the distinction between orbit and paranasal sinus is unclear, the site selected should be paranasal sinus as it is the more likely primary site and requires more aggressive therapy. A primary arising in the sphenoid sinus (rare) may extend inferiorly to involve the nasopharynx
Nasopharynx	This refers to the superior portion of the pharynx which is bounded anteriorly by the back of the nasal septum, superiorly by the sphenoid sinus, inferiorly by a level corresponding to the soft palate and laterally and posteriorly by the pharyngeal walls
Infratemporal fossa/pterygopalatine and parapharyngeal area	This refers to the tissues bounded laterally by the medial lobe of the parotid gland and medially by the pharynx. Large tumours in this region may extend through the parotid gland and present as a mass of the lateral face, sometimes even extending to the cheek. Where there is doubt as to the primary, the parameningeal designation should be chosen as it confers more aggressive treatment. The superior boundary of this tissue volume is the base of the skull just under the temporal lobe, hence the term “infratemporal”. The distinction between this and the parapharyngeal area is academic
Orbital tumours with bone erosion	Tumours arising in the orbit but with intracranial extension or important bone erosion are included in the parameningeal group
In addition the following	• Tumours involving vessels or nerves with direct intracranial connection (e.g., internal carotid and vertebral arteries, optic, trigeminal and facial nerves) • All intracranial and intraspinal tumours (but tumours arise from the paraspinal muscles with intraspinal extension should be designated as paraspinal, see “Other site” definition) • All tumours with cranial nerve paresis (excluding parotid tumours with facial nerve palsy) • Cerebrospinal fluid Tumour cell-positive patients
Non-parameningeal	
Scalp	This site includes primaries arising apparently in, or just below the skin of all tissues of the face and head that are not otherwise specified below. This usually means the scalp, external ear and pinna, the nose and the forehead, but not the eyelids or cheek
Parotid	The parotid gland lies just in front of, and under, the ear and may surround both sides of the posterior aspect of the ascending ramus of the mandible. As noted above, large primaries in the infratemporal fossa may erode through the parotid. A true parotid primary should not, on radiographic studies, reveal a mass in the infratemporal fossa
Oral Cavity	This includes the floor of the mouth, the buccal mucosa, the upper and lower gum, the hard palate and the oral tongue (that portion of the tongue anterior to the circumvallate papillae). A primary arising in the buccal mucosa may be impossible to distinguish from one arising in the cheek, but the distinction is academic. This would also include those lesions arising in or near the lips
Larynx	This refers to primaries arising in the subglottic, glottic or supraglottic tissues. Tumours of the aryepiglottic folds may be impossible to distinguish from the hypopharynx, but the distinction is academic
Oropharynx	This includes tumours arising from the anterior tonsillar pillars, the soft palate, the base of the tongue, the tonsillar fossa, and oropharyngeal walls. Tumours arising in the parapharyngeal space may indent the oropharyngeal wall. In this circumstance, the primary should be considered parameningeal. If the mucosa of the oropharynx actually contains visible tumour as opposed to being displaced by it, the primary would be oropharynx. Primaries arising in the tongue base, soft palate or tonsillar region may extend into the oral cavity. The oropharynx designation is preferred
Cheek	This refers to the soft tissues of the face that surrounds the oral cavity. Tumours arising in the parotid may invade the cheek. As noted above, the distinction between this and the buccal mucosa is academic
Hypopharynx	This refers to the pyriform sinus and may be difficult to distinguish from larynx although the designation is academic
Thyroid and parathyroid	Primaries arising in these two sites are exceedingly rare, if they exist at all, and should these organs be involved, it would more likely be from a primary arising in an adjacent structure such as the neck or, rarely, the trachea
Neck	This refers to the soft tissues of the lateral neck between the mastoid tip and the clavicle. It does not include those medial structures such as hypopharynx and larynx noted above. Unfortunately, this site overlaps with the designation “paraspinal” included under the site group “trunk”. Primaries arising in the neck can and frequently do behave as a paraspinal primary with direct invasion into the spinal extradural space, especially if posteriorly placed
Genito urinary bladder/prostate	
Bladder	Our criteria for identifying the bladder as a primary site has included the appearance of tumour within the bladder cavity, which can be biopsied under cystoscopy or occasionally at laparotomy. We do not recognize as primary bladder tumours those that simply displace the bladder or distort its shape. The latter are ordinarily primary pelvic tumours, unless otherwise specified
Prostate	It is important to differentiate true prostatic tumours from pelvic tumours
Bladder/prostate	In approximately 20% of boys with bladder or prostatic tumours, the precise site cannot be determined even at autopsy. The histologic features are similar. Although it is desirable to have an indication of the “most probable” site from the institution, and one should try to get this, it may not be possible
Genito urinary non-bladder/prostate	
Paratesticular	The tumours arise from mesenchymal elements of the spermatic cord, epididymis and testicular envelopes, producing painless scrotal masses
Testis	This designation is wrong because the tumours arise from paratesticular structures and may invade the testis
Uterus	Tumour in this primary site may be difficult to differentiate from a primary vaginal site, because a tumour originating in the uterus (corpus or cervix) may fill the vagina. After a therapeutic response, the distinction is usually clear. In general, there is a wide separation of age range between these two groups, with the vaginal cases occurring in infancy or early childhood and uterine primaries in adolescents or young adults
Vagina	A patient with a primary vaginal lesion must have evidence of a visible tumour on the vaginal surfaces which can be biopsied through the vagina. Displacement or distortion of the vagina is not sufficient
Vulva	Primary lesions in this site arise in the labia minora or majora
Extremities	
Hand	Refers to the area from the top of the fingers to the wrist
Forearm	Refers to the area from the wrist to the elbow joint
Arm	Refers to the area from the elbow joint to the shoulder joint. Tumours arising in the axilla are considered as extremity lesions
Shoulder	The posterior aspect of the shoulder, i.e., the scapular area, is an extremity site
Foot	Refers to the area from the toes to the ankle
Leg	Refers to the area from the ankle to the knee
Thigh	Refers to the area from the knee to the hip joint
Buttocks	These are extremity lesions
Other	
Thorax	Includes tumours arising in the following sites: a) Thoracic wall: includes tumours arising from the thoracic muscles and the parietal pleura b) Mediastinum: occasionally a primary rhabdomyosarcoma may arise from trachea, heart or nearby areas c) Lung: includes tumours arising from the lung parenchyma, bronchus and visceral pleura d) Breast e) Diaphragm
Abdomen	Includes tumours arising in the following sites: a) Abdominal wall (including lumbar or lumbo-sacral wall): This refers to the anterior abdominal wall from the inferior costal margins superiorly to the inguinal ligaments and symphysis pubis, inferiorly, and extends laterally between the costal margin and posterior iliac crests to the paraspinal region b) Liver True liver rhabdomyosarcoma are less frequent than bile duct tumours c) Bile duct Bile duct is a specific site and can be recognised as such at surgery. This might also be called “choledochus” or “biliary tract”. There is probably no way one can distinguish an intrahepatic bile duct site from a primary liver site except by examining the excised specimen d) Pancreas e) Bowel f) Abdomen The term “abdominal” refers to tumours arising in the intraperitoneal cavity, when a specific organ of origin such as liver, bile duct, pancreas or intestine cannot be determined g) Retroperitoneum The term “retroperitoneal” is reserved for those posteriorly situated abdominal tumours in which there does not seem to be a more specific site. Tumours in a retroperitoneal site are in the posterior aspect of the abdomen and/or pelvis. The term “psoas” as a site is not very specific, as the muscle extends through the posterior lower abdomen, pelvis and into the leg
Paraspinal	When tumours are adjacent to the vertebral column or arising from the paraspinal muscles. This designation is preferable to “abdominal wall”, “trunk” or “neck”. They often show an intraspinal component and this should be specified
Pelvis	It is difficult to define the site of origin when there is a large tumour in the abdomen. The “pelvis” designation is reserved for lesions involving the lower part of the abdomen when no more specific site is appropriate
Perianal	These sites are ordinarily “perirectal” or “perianal”. They are distinguished with difficulty from perineal and vulval sites; but the latter distinction is important
Perineum	This includes the area between the anus and the scrotum in boys and the labia in girls. It extends anteriorly to the base of the scrotum in boys and to the introitus in girls. It must be distinguished from labial and perianal sites
