# Correction to: Imaging in rhabdomyosarcoma: a patient journey

**DOI:** 10.1007/s00247-023-05642-5

**Published:** 2023-03-17

**Authors:** Isabelle S. A. de Vries, Roelof van Ewijk, Laura M. E. Adriaansen, Anneloes E. Bohte, Arthur J. A. T. Braat, Raquel Dávila Fajardo, Laura S. Hiemcke‑Jiwa, Marinka L. F. Hol, Simone A. J. ter Horst, Bart de Keizer, Rutger R. G. Knops, Michael T. Meister, Reineke A. Schoot, Ludi E. Smeele, Sheila Terwisscha van Scheltinga, Bas Vaarwerk, Johannes H. M. Merks, Rick R. van Rijn

**Affiliations:** 1 Princess Máxima Center for Pediatric Oncology, Utrecht, the Netherlands; 2 Department of Radiology and Nuclear Medicine, University Medical Centre Utrecht, Utrecht, the Netherlands; 3 Department of Radiotherapy, University Medical Centre Utrecht, Utrecht, the Netherlands; 4 Department of Pathology, University Medical Centre Utrecht, Utrecht, the Netherlands; 5 Department of Otorhinolaryngology, University Medical Centre Utrecht, Utrecht, the Netherlands; 6 Oncode Institute, Utrecht, the Netherlands; 7 Department of Head and Neck Oncology and Surgery, The Netherlands Cancer Institute (NCI), Amsterdam, the Netherlands; 8 Department of Oral and Maxillofacial Surgery, Amsterdam UMC, University of Amsterdam, Amsterdam, the Netherlands; 9 Department of Paediatrics, Amsterdam UMC – Emma Children’s Hospital, University of Amsterdam, Amsterdam, the Netherlands; 10 Department of Radiology and Nuclear Medicine, Amsterdam UMC – Emma Children’s Hospital, University of Amsterdam, Suite C1‑423.1, Meibergdreef 9, 1105AZ Amsterdam, the Netherlands

**Correction to** **:** **Pediatric Radiology (2023)**

**https://doi.org/10.1007/s00247-023-05596-8**

The original published online version stated:

"For primary tumour size, 2-dimensional (D) measurements according to Response Evaluation Criteria In Solid Tumours (RECIST) 1.1 are recommended in the European guideline and used for international studies [17, 24]."

This was an error, and the sentence should read:

"For primary tumour size, 1-dimensional (D) measurements according to Response Evaluation Criteria In Solid Tumours (RECIST) 1.1 are recommended in the European guideline and used for international studies [17, 24]."

The original article has been corrected.

